# OGT-mediated O-GlcNAcylation of MAGI1 exacerbates high glucose-triggered inflammation and dedifferentiation of vascular smooth muscle cells by activating the PI3K/AKT pathway

**DOI:** 10.1186/s41065-026-00643-4

**Published:** 2026-01-16

**Authors:** Li Wen, Ruijiang Dai, Shuang Yu, Houzhi Yu

**Affiliations:** 1 Department of Neurology, Shandong Provincial Hospital Affiliated to Shandong First Medical University, Jinan, Shandong 250021 China; 2 Clinical Medicine (Sino-Foreign Cooperative Education), Chongqing Medical University, Chongqing, 401331 China; 3 Department of Cardiology, Zhoucun People’s Hospital of Zibo, Zibo, Shandong 255330 China; 4 Department of Cardiology, Shandong Provincial Hospital Affiliated to Shandong First Medical University, No.324, Jingwuweiqi Road, Jinan, Shandong 250021 China

**Keywords:** Diabetes mellitus, Vascular smooth muscle cells, O-GlcNAc transferase, Membrane-associated guanylate kinase with an inverted domain structure-1, O-GlcNAc modification

## Abstract

**Background:**

Vasculopathy is a vital complication of diabetes mellitus (DM), and the dysfunction of vascular smooth muscle cells (VSMCs) is a central event in its pathogenesis. O-GlcNAc transferase (OGT), the enzyme catalyzing O-GlcNAcylation, is implicated in diabetic complications, yet its specific role in VSMC dysfunction remains poorly defined. This study aimed to elucidate the function of OGT and its downstream signaling in high glucose (HG)-induced VSMC injury.

**Methods:**

A cellular model of DM was established by treating human VSMCs with HG. Expression analysis was performed by RT-qPCR and western blot, respectively. Cell viability, proliferation, and migration/invasion were assessed using CCK-8, EdU, and transwell assays. Inflammatory cytokine secretion was measured by ELISA. A diabetic mouse model was established by streptozotocin (STZ) to validate the in vivo relevance.

**Results:**

Membrane-associated guanylate kinase with an inverted domain structure-1 (MAGI1) was up-regulated in DM patients and HG-induced VSMCs. Functionally, MAGI1 knockdown attenuated HG-induced VSMC dysfunction, suppressing proliferation, migration, invasion, inflammatory response, and dedifferentiation. Conversely, MAGI1 overexpression exacerbated these pathological phenotypes. Mechanistically, MAGI1 activated the PI3K/AKT signaling pathway in HG-induced VSMCs. Moreover, OGT mediated the O-GlcNAcylation and stability of MAGI1. Knockdown of OGT alleviated HG-induced VSMC dysfunction and inhibited the PI3K/AKT pathway by reducing MAGI1 expression. In vivo, OGT deficiency ameliorated kidney injury and systemic inflammation in STZ-induced diabetic mice.

**Conclusion:**

This study demonstrates that OGT promotes MAGI1 expression through O-GlcNAc modification to drive VSMC dysfunction. This study not only delineates a previously unrecognized mechanism but also identifies the OGT/MAGI1 axis as a potential therapeutic target for preventing vascular complications in diabetes.

**Supplementary Information:**

The online version contains supplementary material available at 10.1186/s41065-026-00643-4.

## Introduction

Diabetes mellitus (DM) represents a global health crisis characterized by chronic hyperglycemia [1]. This metabolic disorder not only significantly impairs patients’ quality of life but also predisposes them to a spectrum of debilitating complications [2]. Among the most prevalent and severe consequences of DM is diabetic vasculopathy, a term encompassing both microvascular (e.g., retinopathy, nephropathy) and macrovascular (e.g., accelerated atherosclerosis) complications, which collectively represent the leading cause of morbidity and mortality in the diabetic population [3, 4]. Current therapeutic strategies for diabetic vasculopathy primarily focus on stringent glycemic control and management of concomitant risk factors, such as hypertension and dyslipidemia. While essential, these approaches often fail to fully halt the progression of vascular damage, underscoring the critical need for novel interventions targeting the underlying pathophysiological mechanisms. Within this context, vascular smooth muscle cells (VSMCs), the principal cellular components of the vascular media, have emerged as critical players in the initiation and progression of diabetic vascular complications. Under diabetic conditions such as hyperglycemia and insulin resistance, VSMCs undergo excessive proliferation, migration, and phenotypic switching, ultimately contributing to vascular dysfunction and atherosclerosis [3]. The molecular mechanisms orchestrating this phenotypic switch are complex and multifactorial, involving hyperglycemia-induced oxidative stress, aberrant activation of inflammatory signaling pathways (e.g., NF-κB), and dysregulation of key transcription factors [5, 6]. Recent studies have also highlighted the involvement of epigenetic modifications and non-coding RNAs in modulating VSMC behavior [7]. Consequently, exploring the precise mechanisms of VSMC dysfunction is a crucial step toward identifying novel molecular targets and developing molecularly targeted therapeutics in DM.

Membrane-associated guanylate kinase with an inverted domain structure-1 (MAGI1) is a pivotal scaffolding protein that belongs to the MAGUK family, predominantly known for its role in maintaining cell-cell adhesion and polarity by organizing protein complexes at specialized membrane domains [8]. Beyond its structural functions, MAGI1 has emerged as a significant regulator of intracellular signaling pathways, and its dysregulation has been implicated in various human diseases, including cancer and neurological disorders [9, 10]. However, the role of MAGI1 in regulating VSMC function under DM conditions is unknown.

O-Linked β-N-acetylglucosamine (O-GlcNAc) represents a highly dynamic monosaccharide post-translational modification, with tight relation to the regulation of hemostasis, inflammation and tumors [11]. O-GlcNAc modification (O-GlcNAcylation) of protein is associated with vascular dysfunction in DM and diabetic complications [12–15]. O-GlcNAc transferase (OGT), a pivotal enzyme for O-GlcNAcylation, reveals the significant mediation in VSMC dedifferentiation of hyperglycemia [16]. However, the role and mechanism of the OGT-MAGI1 axis in VSMC dysfunction remain elusive.

Thus, the current study aimed to explore a novel regulatory mechanism associated with OGT and MAGI1 in high glucose (HG)-induced VSMCs. A range of cell functions including proliferation, migration, invasion, inflammation and dedifferentiation were explored. OGT-mediated O-GlcNAcylation of MAGI1 in VSMC dysfunction was considered as a research emphasis.

## Materials and methods

### Bioinformatics

Gene expression data were obtained from the GSE25724 dataset on the NCBI GEO database (https://www.ncbi.nlm.nih.gov/geo/query/acc.cgi?acc=GSE25724, accessed on 16 October 2023). This dataset was selected as it provides transcriptomic profiles of pancreatic islets from well-characterized cohorts of human donors with and without type 2 diabetes. Differentially expressed genes were identified using a threshold of *P* < 0.05 and |log2(FoldChange)| > 0.5. The potential O-GlcNAc sites of MAGI1 were predicted using the Oglcnac database v2.0 (https://www.oglcnac.mcw.edu/, accessed on 8 March 2024).

### Patients and samples

A total of 30 patients with DM and 30 age- and sex-matched healthy controls were enrolled from Shandong Provincial Hospital Affiliated to Shandong First Medical University. All participants provided written informed consent prior to inclusion. Diabetic patients were diagnosed according to standard clinical criteria. Patients with DM were excluded if they had used medications known to influence O-GlcNAcylation (e.g., metformin) or vascular function (e.g., statins) within three months prior to enrollment, thereby minimizing potential pharmacological confounding in the analysis of serum MAGI1 and OGT levels. The control subjects were confirmed to have normal glucose tolerance and no history of diabetes or other metabolic diseases. The two groups were matched for age (± 3 years) and sex to minimize potential confounding factors. Fresh blood samples were collected, and serum was obtained by centrifugation. Serum samples were stored at -80°C for subsequent analysis. This research was performed based on the approval by the Ethics Committee of Shandong Provincial Hospital Affiliated to Shandong First Medical University.

### Cell culture and treatment

Human VSMCs were purchased from TongWei Biotechnology Co., LTD. (Shanghai, China). VSMCs were cultured in DMEM enriched with 10% fetal bovine serum and 1% penicillin-streptomycin solution (Gibco, Carlsbad, CA, USA). Cell culture was performed in the 37 °C incubator containing 5% CO_2_. DM model was established in VSMCs by exposure to 30 mM glucose (HG), and 5.5 mM glucose (normal glucose) treatment was employed as the control group. Additionally, an osmotic control group (5.5 mM glucose + 24.5 mM mannitol) was used to ensure the glucose-specific biological effects in crucial experiments.

### Transient transfection

VSMCs were stably transfected with plasmids through Lipofectamine™ 3000 Kit (Invitrogen, Carlsbad, CA, USA), following the operating instructions of the manufacturer. In regard to plasmids, short hairpin RNA (shRNA) plasmids (shNC, shMAGI1, shOGT) and pcDNA plasmids (oeNC, oeMAGI1) were respectively provided by GenePharma (Shanghai, China) and RIBOBIO (Guangzhou, China).

### Real-time quantitative PCR (RT-qPCR)

Reverse transcription was implemented by PrimeScript™ FAST RT reagent Kit (Takara, Beijing, China) after total RNA extraction using Trizol (Invitrogen). Thereafter, cDNA amplification reaction was performed through TB Green^®^ Premix Ex Taq™ II reagent (Takara). All procedures were performed in strict accordance with the specifications. The primer sequences were exhibited in Table 1. MAGI1 mRNA level was analyzed using the 2^−∆∆Ct^ method, with β-actin as the normalization control.

**Table 1:** Primer sequences used for RT-qPCR

Name	Primer sequences (5’-3’)	
MAGI1	Forward	GCCACTGCAGGGATCAAGTA
	Reverse	TCAGGCAGCTGTGACTCTTG
ACTA2	Forward	GTACCCAGGCATTGCTGACA
	Reverse	GAGGCGCTGATCCACAAAAC
LMOD1	Forward	GGTCTGTTCCTCAGCCAGTC
	Reverse	CTGCCTTCCTTCGCCTGTAA
PCNA	Forward	AAAGATGCCGTCGGGTGAAT
	Reverse	TGGTTACCGCCTCCTCTTCT
β-actin	Forward	GGATTCCTATGTGGGCGACGA
	Reverse	GCGTACAGGGATAGCACAGC

### Western blot

After proteins were acquired through the lysis of RIPA buffer (Thermo Fisher, Waltham, MA, USA), electrophoresis was carried out with 50 µg proteins of each sample. Subsequently, proteins on the gels were transferred to PVDF membranes (Thermo Fisher) followed by protein blocking in western blocking buffer (Beyotime, Shanghai, China). Then, the membranes were washed and incubated with the primary antibody at 4℃ overnight and the secondary antibody at room temperature for 1 h. Antibody information was as below: anti-MAGI1 (Abcam, Cambridge, UK, #ab255611), anti-OGT (Abcam, #ab177941), anti-ACTA2 (Thermo Fisher, #23GB4745), anti-LMOD1 (Abcam, #ab244415), anti-PCNA (Abcam, #ab18197), anti-O-GlcNAc (Abcam, #ab320076), anti-PI3K (Abcam, #ab191606), anti-p-PI3K (Abcam, #ab278545), anti-AKT (CST, Boston, MA, USA, #9271), anti-p-AKT (CST, #9271), anti-β-actin (Abcam, #ab8227, 1:2,000), the goat anti-rabbit secondary antibody (Abcam, #ab205718, 1:10,000). Afterwards, protein signals were determined using ECL Substrate (Thermo Fisher) followed by level analysis in Image J software.

### Cell counting Kit-8 (CCK-8) assay

VSMCs were cultured for 24 h, then received HG treatment and plasmid transfection. For cell viability detection, 10 µL CCK-8 solution (Beyotime) was added to cells of each well after cell medium was removed. Absorbance at 450 nm was examined through a microplate reader.

### Ethynyl-2’-deoxyuridine (EdU) assay

The proliferation ability was determined using EdU Cell Proliferation Detection Kit (SUNNCELL, Wuhan, China). VSMCs were stained with EdU, according to the supplied instruction, and then DNA was dyed with DAPI in the dark, followed by fluorescence detection through a fluorescence microscope (Olympus, Tokyo, Japan). EdU positive cells = EdU + DAPI merged cells.

### Transwell assay

Transwell chamber (Corning Inc., Corning, NY, USA) coated with or without Matrigel (Corning Inc.) was utilized for ability analysis of cell invasion and migration, respectively. The chambers were placed in 24-well plates and inoculated with VSMCs suspended in serum-free medium, while complete medium was added to the lower chambers. Cell incubation at 37°C was conducted for 24 h, followed by cell fixation and staining with 4% paraformaldehyde (Beyotime) and crystal violet (Beyotime). Cells on the upper surface of the membrane were gently removed with cotton swabs. The migrated or invaded cells on the lower membrane surface were imaged and counted in five random fields per membrane under a light microscope (×200 magnification). Counting was performed independently by two researchers who were blinded to the experimental groups to ensure objectivity. The average value from the five fields was calculated for each replicate, and three independent experiments were performed.

### Enzyme-linked immunosorbent assay (ELISA)

Cell supernatant was harvested from VSMCs after HG treatment and different transfection. The concentrations (pg/mL) of tumor necrosis factor-alpha (TNF-α) and interleukin-1beta (IL-1β) were tested via the corresponding ELISA Kits (Invitrogen) as per the users’ guidance.

### Immunoprecipitation

VSMCs were transfected with HG, HG + shNC or HG + shOGT. Then protein extracts (2 mg) were incubated with anti-OGlcNAc and protein A/G agarose beads at 4 °C overnight. Samples were collected from beads, followed by western blot detection for MAGI1.

### Animal models

C57BL/6 male mice (*n* = 27, SiPeiFu, Beijing, China) aged 6 to 8 weeks were raised and cared in line with the Management and Use Guidelines of Laboratory Animals published by NIH. Primary mouse aortic VSMCs were isolated from three male C57BL/6J mice following a standard enzymatic digestion protocol. Briefly, after euthanasia, the thoracic aorta was aseptically excised, cleaned of adherent fat and connective tissue, and digested with 1 mg/mL collagenase type II for 30 min at 37 °C. The adventitia and endothelium were mechanically removed. The remaining medial layer was minced and further digested in 0.5 mg/mL collagenase type I for 2 h. The released cells were collected by centrifugation, resuspended in DMEM supplemented with 10% FBS and 1% penicillin-streptomycin, and cultured at 37°C in a humidified 5% CO_2_ incubator. Cells from passages 3–5 were used for transfection and in vivo experiments.

Mice (*n* = 24) were randomly divided into four groups: Control (*n* = 6), STZ (*n* = 6), STZ + shNC (*n* = 6), STZ + shOGT (*n* = 6). To establish DM model, mice were fed a 60% high‑fat diet for 8 weeks, followed by intraperitoneal injection of STZ (50 mg/kg/day) for five consecutive days as previously reported [17]. The mice in the control group were fed with standard calorie diet and injected with citrate buffer. Diabetes induction was confirmed three days after the last STZ injection: mice with blood glucose > 16.7 mM and HbA1c > 6.5% on three consecutive days were considered diabetic. Two weeks after confirmation, 2 × 10^6^ mouse aortic VSMCs transfected with shNC or shOGT (transfection efficiency > 70%, viability > 95%) suspended in 100 µL PBS were implanted into the right common carotid artery via carotid cannulation. Vascular function was assessed 4 weeks after transplantation.

Blood samples were collected for western blot and ELISA detection. In addition, HE staining and Masson staining were performed to observe the pathogenic change of kidney tissues. This animal research was ratified by the Animal Care Committee of Shandong Provincial Hospital Affiliated to Shandong First Medical University. This study was prepared in compliance with the ARRIVE guidelines 2.0. The sample size of six mice per group was determined a priori based on a power analysis using G*Power software (version 3.1). The analysis was conducted with an alpha (α) level of 0.05, a statistical power (1-β) of 80%, and an expected large effect size (f = 0.8) derived from preliminary data. This sample size ensures a high probability of detecting statistically significant differences in key vascular parameters among the groups.

### Statistical analysis

Data were all indicated as mean ± SD, then SPSS and GraphPad Prism were applied for data analysis and graph production. For two groups, Student’s *t*-test was employed for difference analysis. Difference among three groups and more than three groups was assessed via analysis of variance (ANOVA). In all analyses, *P* < 0.05 was defined as statistically significant. The assays were independently repeated three times with three parallels in each time.

## Results

### MAGI1 was highly expressed in DM patients and HG-induced VSMCs

**Fig. 1:**
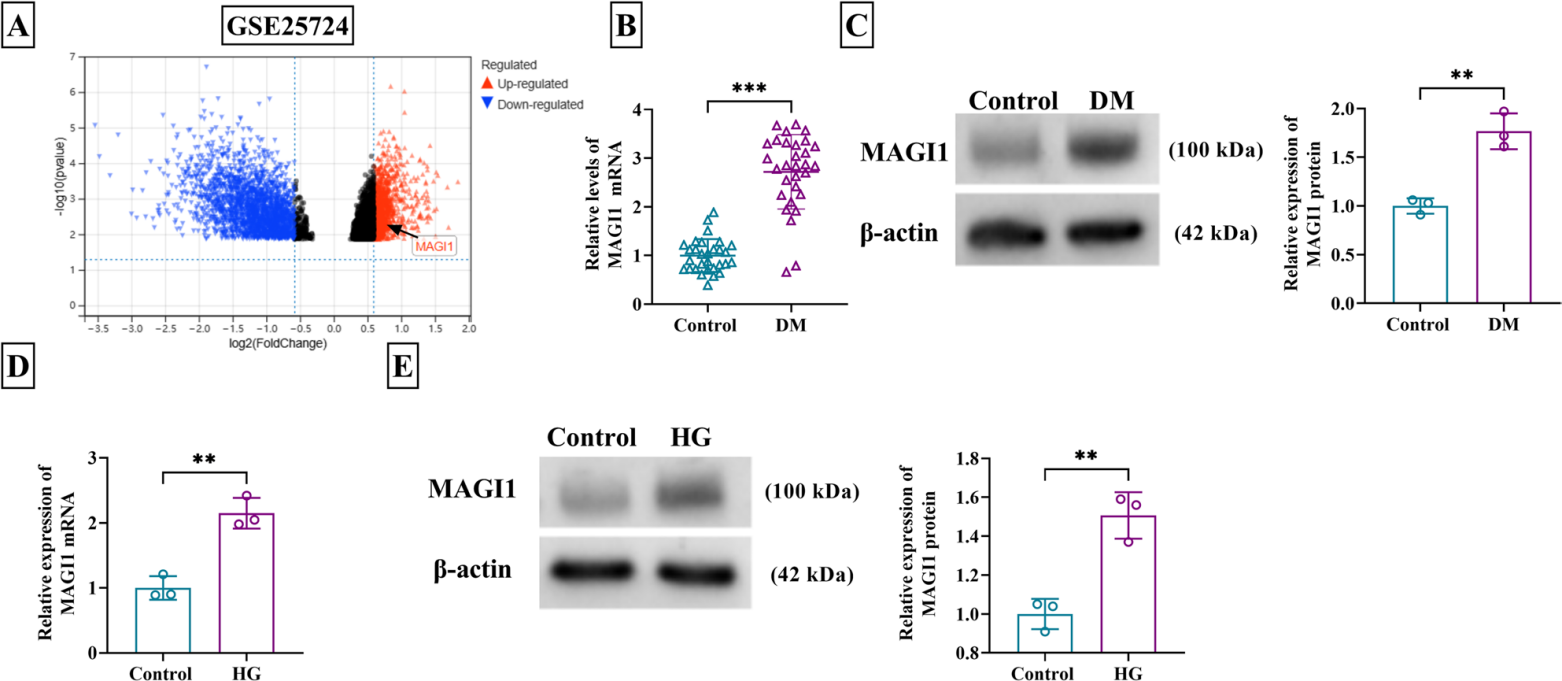
MAGI1 was highly expressed in DM patients and HG-induced VSMCs. **A** GSE25724 dataset for MAGI1 expression in islet samples from DM patients. **B-C** MAGI1 mRNA and protein detection was conducted via RT-qPCR and western blot. **D-E** VSMCs were treated with HG (30 mM glucose) and control (5.5 mM glucose). RT-qPCR and western blot were applied to examine MAGI1 mRNA and protein levels. ^**^P < 0.01, ^***^P < 0.001

In the GSE25724 dataset, MAGI1 was up-regulated in type 2 diabetic human islets compared to non-diabetic islet samples (Fig. 1A). Then, the detection by RT-qPCR and western blot affirmed the high mRNA and protein levels of MAGI1 in serum samples from DM patients (Fig. 1B-C). Additionally, MAGI1 expression was significantly associated with estimated glomerular filtration rate (eGFR), ankle-brachial index (ABI), and early treatment diabetic retinopathy study (ETDRS) of DM patients (Table 2). In vitro, cell model of DM was induced by HG treatment to VSMCs. Both MAGI1 mRNA and protein levels were increased in HG-treated VSMCs with respect to control group (Fig. 1D-E). Collectively, these results demonstrated that MAGI1 was upregulated in DM patients and in an HG-induced cellular model.

**Table 2:** Correlation of MAGI1 expression with the crucial clinicopathologic features of DM patients (*n* = 30)

Parameters	*n* = 30	MAGI1 expression		*p*-value
		High *n* = 15	Low *n* = 15	
eGFR(ml/min/1.73 m²)				*P < 0.05
≥ 60	18	5	13	
< 60	12	10	2	
ABI				*P < 0.05
≤ 0.9	13	11	2	
> 0.9	17	4	13	
ETDRS				*P < 0.05
≥ 43	14	10	4	
< 43	16	5	11	

*eGFR* Estimated glomerular filtration rate, *ABI* Ankle-brachial index, *ETDRS* Early treatment diabetic retinopathy study

^*^P < 0.05, statistically significant

### MAGI1 knockdown inhibited HG-induced proliferation, migration, invasion, inflammation, and dedifferentiation of VSMCs via mediating PI3K/AKT pathway

**Fig. 2:**
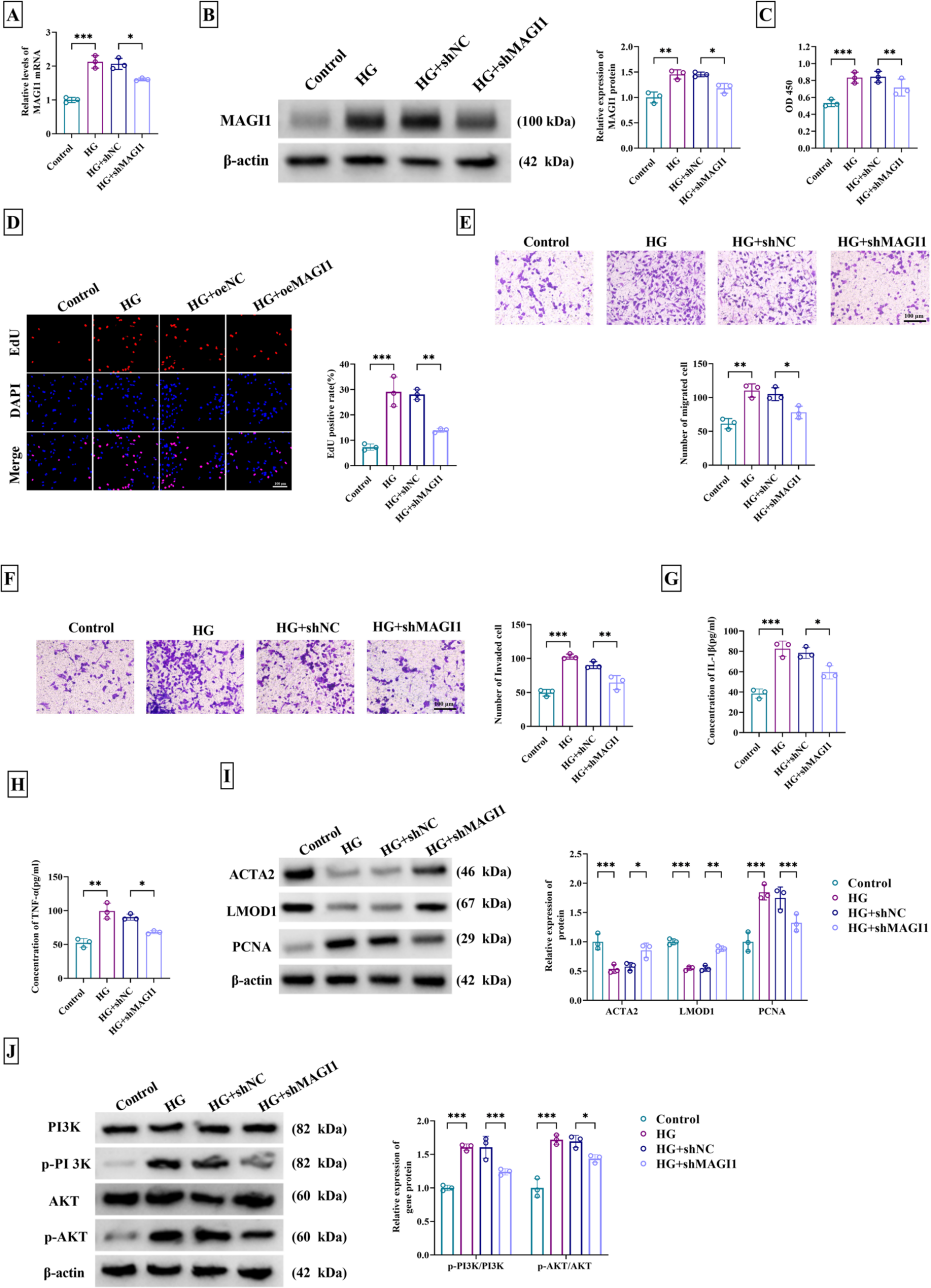
MAGI1 knockdown inhibited HG-induced proliferation, migration, invasion, inflammation, and dedifferentiation of VSMCs via mediating PI3K/AKT pathway. VSMCs were treated with control, HG, HG + shNC, or HG + shMAGI1. **A-B** RT-qPCR for MAGI1 mRNA detection (**A**) and western blot for MAGI1 protein detection (**B**). **C** CCK-8 assay for examination of cell viability. **D** EdU assay for detection of proliferation. **E-F** Transwell assay for assessment of migration (**E**) and invasion (**F**). **G-H** ELISA for determination of pro-inflammatory cytokines IL-1β (**G**) and TNF-α (**H**). **I** Western blot for level analysis of contractility markers (ACTA2, LMOD1) and proliferation marker PCNA. **J** Western blot for level detection of PI3K, p-PI3K, AKT, and p-AKT. ^*^P < 0.05, ^**^P < 0.01, ^***^P < 0.001

To investigate the function of MAGI1, loss-of-function assays were performed using shRNA-mediated knockdown. As the results of RT-qPCR and western blot, shMAGI1 inhibited HG-upregulated expression of MAGI1 in VSMCs, indicating excellent knockdown of MAGI1 (Fig. 2A-B). CCK-8 and EdU assays demonstrated that HG treatment enhanced cell viability (Fig. 2C) and proliferation (Fig. 2D), which was subsequently reversed by silencing of MAGI1. Transwell assay was performed to assess cell motility. Cell migration (Fig. 2E) and invasion (Fig. 2F) were evidently promoted in HG-treated VSMCs, then these effects were ameliorated following co-treatment with shMAGI1. Also, ELISA for IL-1β and TNF-α suggested that MAGI1 downregulation alleviated the promoting impact of HG on inflammatory reaction (Fig. 2G-H). ACTA2 and LMOD1 are common genes marked VSMC differentiation [18]. Then these proteins and proliferation marker PCNA were detected by western blot. ACTA2 and LMOD1 levels were elevated while PCNA protein expression was decreased in HG + shMAGI1 group compared to the HG-only group, uncovering the inhibition of shMAGI1 for HG-induced VSMC dedifferentiation (Fig. 2I). Besides, HG-mediated upregulation of p-PI3K/PI3K and p-AKT/AKT was restored following shMAGI1 introduction, suggesting the inactivation of PI3K/AKT pathway by MAGI1 knockdown (Fig. 2J). Thus, silencing MAGI1 relieved vascular injury in HG-treated VSMCs via the inhibition of PI3K/AKT pathway.

### Overexpression of MAGI1 contributed to HG-induced VSMC dysfunction by promoting PI3K/AKT pathway

**Fig. 3:**
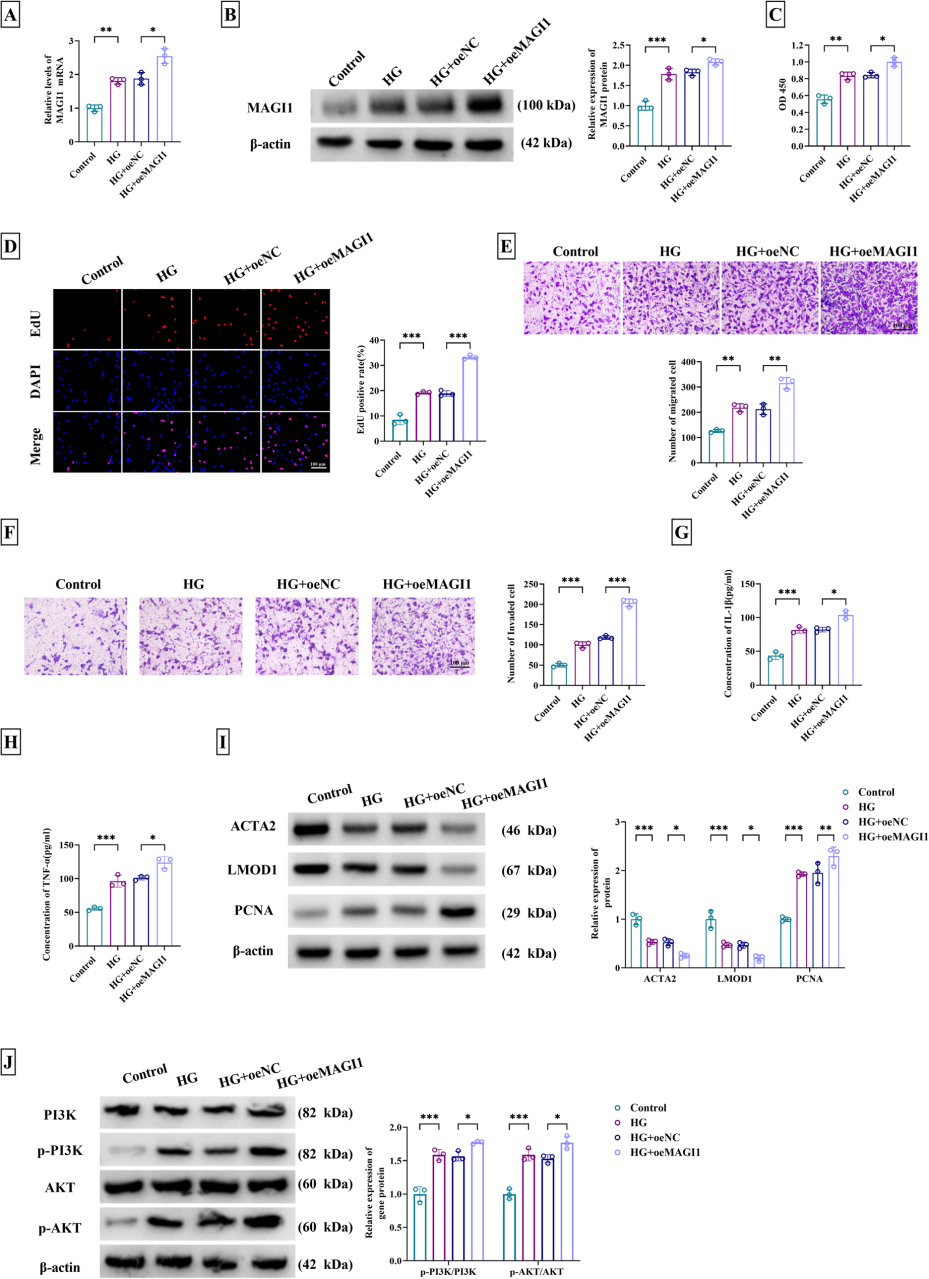
Overexpression of MAGI1 contributed to HG-induced VSMC dysfunction by promoting PI3K/AKT pathway. VSMCs were treated with control, HG, HG + oeNC, or HG + oeMAGI1. **A-B** MAGI1 expression analysis for mRNA (**A**) and protein (**B**) using qPCR and western blot. **C** Cell viability analysis by CCK-8 assay. **D** Cell proliferation detection by EdU assay. **E-F** Examination of migration (**E**) and invasion (**F**) by transwell assay. **G-H** Level detection of IL-1β (**G**) and TNF-α (**H**) via ELISA. **I** The protein determination of contractility markers (ACTA2, LMOD1) and proliferation marker PCNA through western blot. **J** The protein detection of PI3K, p-PI3K, AKT, and p-AKT via western blot.^*^P < 0.05, ^**^P < 0.01, ^***^P < 0.001

Correspondingly, the regulation of MAGI1 overexpression was investigated in VSMCs. MAGI1 overexpression was achieved by transfection of oeMAGI1, which distinctly up-regulated the mRNA and protein levels of MAGI1 in HG-treated VSMCs (Fig. 3A-B). HG-stimulated facilitating influences on viability (Fig. 3C), proliferation (Fig. 3D), migration (Fig. 3E), and invasion (Fig. 3F) were all further exacerbated with the introduction of oeMAGI1. Meanwhile, pro-inflammatory IL-1β and TNF-α levels were much higher in HG + oeMAGI1 group than these in HG + oeNC group (Fig. 3G-H). Additionally, expression increase of MAGI1 intensified the protein level changes of ACTA2, LMOD1, and PCNA caused by HG (Fig. 3I). For PI3K/AKT pathway, MAGI1 overexpression enhanced protein levels of p-PI3K/PI3K and p-AKT/AKT under HG induction (Fig. 3J). Contrary to MAGI1 knockdown, its overexpression in VSMCs accelerated HG-mediated dysfunction through PI3K/AKT activation.

### Silence of OGT reduced the O-GlcNAc level of MAGI1

**Fig. 4:**
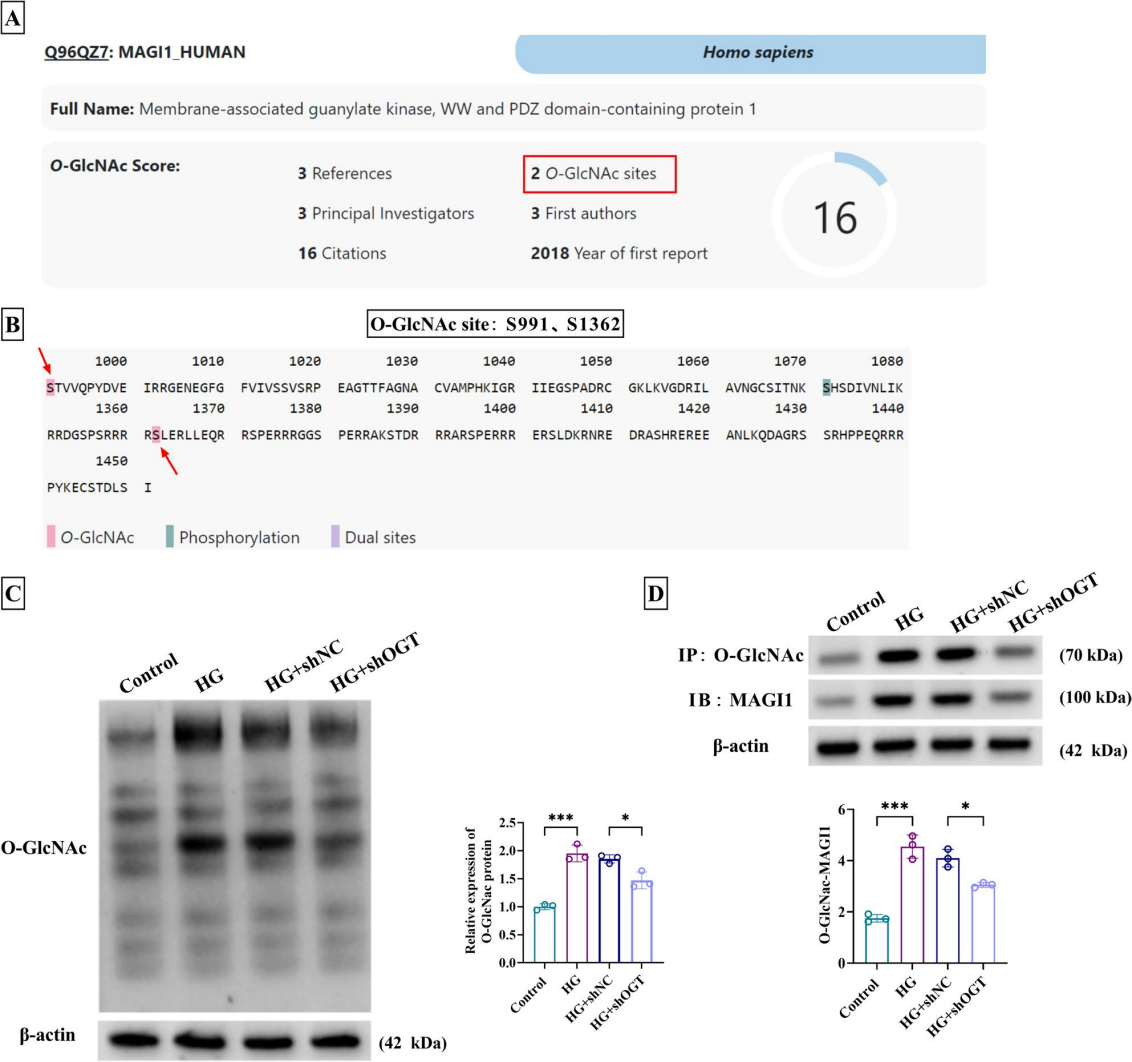
Silence of OGT reduced the O-GlcNAc level of MAGI1. **A** MAGI1 was predicted to have O-GlcNAc sites by oglcnac. **B** The O-GlcNAc sites of MAGI1. **C** O-GLcNAc protein level was measured using western blot in VSMCs of control, HG, HG + shNC, or HG + shOGT group. **D** Immunoprecipitation was performed to analyze the interaction between OGT and MAGI1. ^*^P < 0.05, ^***^P < 0.001

Through the prediction of Oglcnac (https://www.oglcnac.mcw.edu/), it was found that MAGI1 contained the O-GlcNAc sites (Fig. 4A) and two O-GlcNAc sites (S991, S1362) were shown as Fig. 4B. Wild-type MAGI1 (MAGI1-WT) was found to increase the O-GlcNAc protein level, while single mutant MAGI1-S991A or MAGI1-S1362A reduced O-GlcNAc protein and the double mutants (MAGI1-DM) further inhibited O-GlcNAc protein level (Supplementary Fig. 1A). Moreover, single mutant MAGI1-S991A or MAGI1-S1362A partially suppressed cell viability, migration, and the PI3K/AKT pathway activation, and the double mutants (MAGI1-DM) further enhanced these suppressive effects (Supplementary Fig. 1B-1D). The site-directed mutation suggested that O-GlcNAc modification in MAGI1 indeed depends on S991 and S1362 sites. VSMCs with HG induction displayed the increased O-GlcNAc protein expression (Fig. 4C and Supplementary Fig. 2A), while the Osmo group did not affect cell O-GlcNAc protein expression (Supplementary Fig. 2A), demonstrating the effect was due to glucose-specific biological effects. However, VSMCs from the HG + shOGT group hindered the O-GlcNAc level compared with the HG + shNC group (Fig. 4C). Further immunoprecipitation analysis demonstrated that MAGI1 was a target for O-GlcNAc modification, and knockdown of OGT declined the expression of O-GlcNAc-modified MAGI1 when compared with alone HG treatment (Fig. 4D). The above evidence elucidated that OGT could enhance the O-GlcNAc level of MAGI1.

### OGT mediated VSMC dysfunction and affected the PI3K/AKT pathway by upregulating MAGI1

**Fig. 5:**
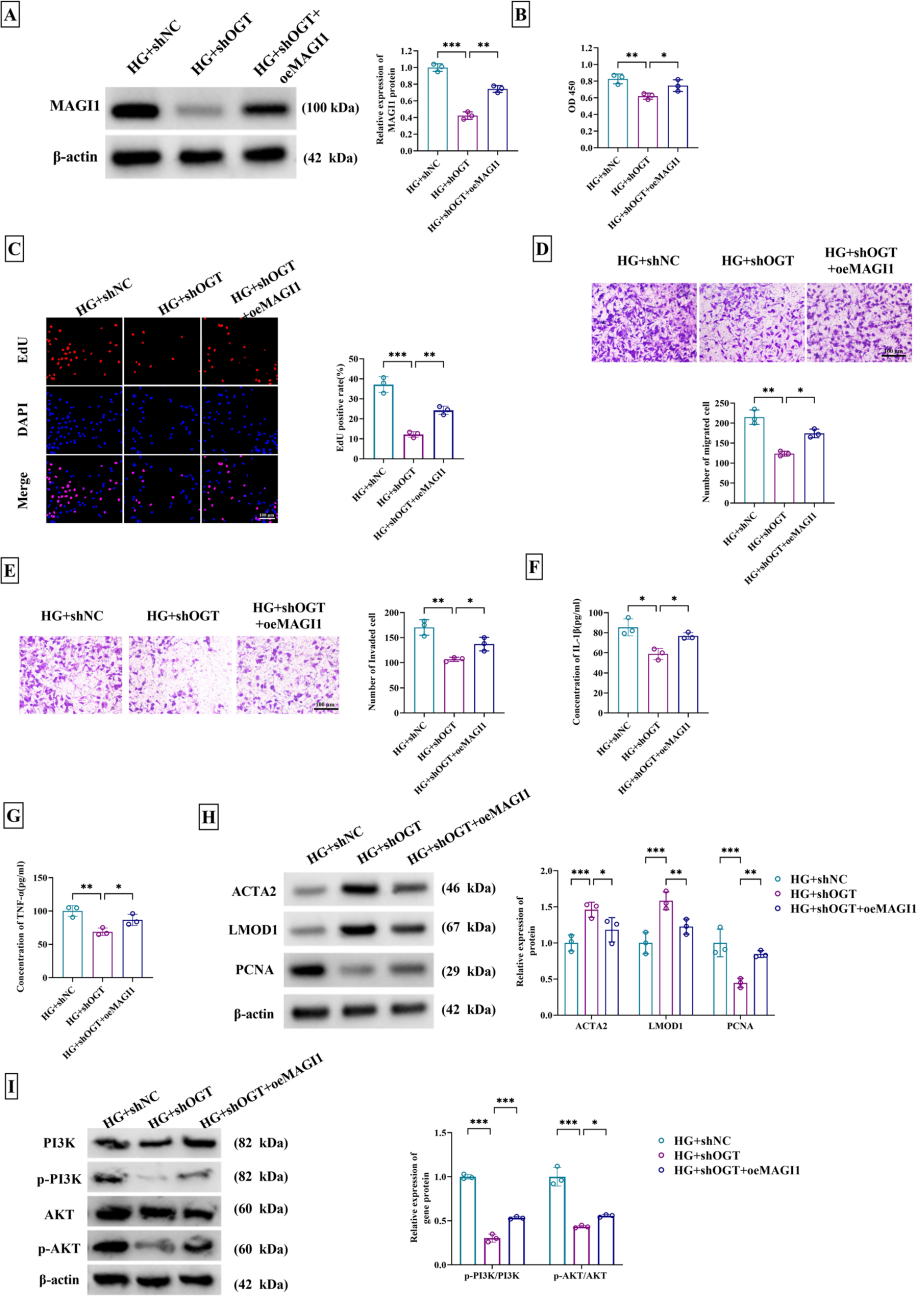
OGT mediated VSMC dysfunction by upregulating MAGI1 to affect PI3K/AKT pathway. Treatment of HG + shNC, HG + shOGT, or HG + shOGT + oeMAGI1 was performed in VSMCs. **A** MAGI1 protein level was measured via western blot. **B** Cell viability was determined using CCK-8 assay. **C** The proliferation capacity was assessed using EdU assay. **D-E** Cell migration (**D**) and invasion (**E**) abilities were estimated by transwell assay. **F-G** IL-1β (F) and TNF-α (**G**) levels were examined through ELISA. **H** Contractility markers (ACTA2, LMOD1) and proliferation marker PCNA were detected via western blot. **I** The protein levels of PI3K, p-PI3K, AKT, and p-AKT were examined using western blot. ^*^P < 0.05, ^**^P < 0.01, ^***^P < 0.001

To explore the involvement of OGT and MAGI1 in vascular injury, VSMCs were co-treated with HG + shOGT + oeMAGI1. MAGI1 protein expression was decreased by shOGT under the treatment of HG, which was significantly reversed after co-treatment with oeMAGI1 (Fig. 5A). Therefore, OGT exhibited a positive effect on MAGI1 expression in HG-stimulated VSMCs. Cellular detection under HG treatment revealed that downregulation of OGT resulted in the prominent inhibition of cell viability (Fig. 5B), proliferation (Fig. 5C), migration (Fig. 5D) and invasion (Fig. 5E), whereas the following MAGI1 overexpression enervated this regulation. As the results of MAGI1 expression increase, shOGT-mediated level reduction of IL-1β and TNF-α was curtailed in HG-treated VSMCs (Fig. 5F-G). Western blot indicated that HG + shOGT + oeMAGI1 group effectively recovered the upregulation of ACTA2 and LMOD1, as well as the downregulation of PCNA, compared to the HG + shOGT group (Fig. 5H). Moreover, p-PI3K/PI3K and p-AKT/AKT expression reduction in OGT-silenced VSMCs upon HG induction was significantly abolished by MAGI1 overexpression (Fig. 5I). Taken together, the regulation of OGT knockdown in HG-induced VSMC dysfunction was achieved by decreasing MAGI1 to inhibit the PI3K/AKT pathway.

In addition, an OGT expression plasmid (OGT-WT) or its mutations in the catalytically active site (OGT-H558A) significantly increased OGT expression in shOGT-transfected VSMCs under HG (Supplementary Fig. 3A). Moreover, OGT-WT, but not OGT-H558A, significantly reversed shOGT-mediated alterations in MAGI1 O-GlcNAc levels, cell viability, and cell migration in VSMCs under HG (Supplementary Fig. 3B-3D).

### OGT affected kidney injury and serum inflammation level in STZ-induced DM mice

**Fig. 6:**
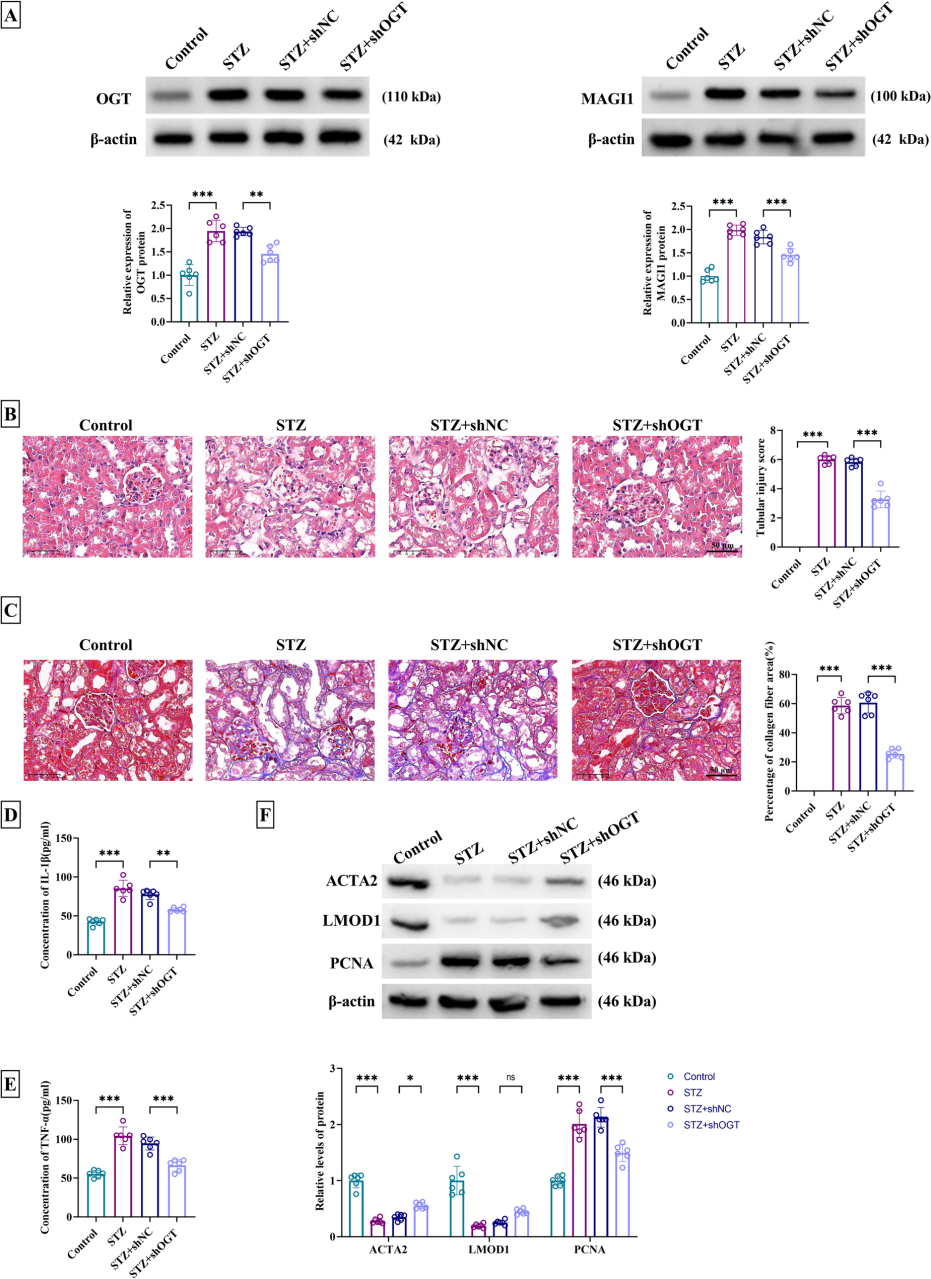
OGT affected kidney injury and serum inflammation level in STZ-induced DM mice by targeting MAGI1. Mice were divided into control, STZ (50 mg/kg), STZ + shNC or STZ + shOGT group. **A** Western blot was used to determine protein levels of OGT and MAGI1 in serum samples. **B-C** H&E staining (**B**) and Masson staining (**C**) of kidney tissues from each group. H&E pathological changes were scored by evaluating tubular injury in five random fields and calculating the sum. 0: none; 1: mild (< 25%); 2: moderate (25–50%); 3: severe (> 50%). The renal fibrosis area was quantified by ImageJ and expressed as a percentage relative to the control group. **D-E** ELISA was performed to examine IL-1β (**D**) and TNF-α (**E**) in serum samples. **F** Contractility markers (ACTA2, LMOD1) and proliferation marker PCNA in serum samples were detected using western blot. ^*^P < 0.05, ^**^P < 0.01, ^***^P < 0.001

For further research in vivo, STZ was used to establish DM model in mice. Serum samples from STZ group exhibited the obvious upregulation of OGT and MAGI1 proteins compared to the control group, and these levels were evidently suppressed in STZ + shOGT group relative to STZ + shNC group (Fig. 6A). HE staining revealed significant tubular injury after STZ induction, while this kidney injury was ameliorated by shOGT (Fig. 6B). Masson staining showed STZ-caused increase of collagen fiber area was relieved following knockdown of OGT (Fig. 6C). For inflammatory reaction in vivo, STZ-induced level increase of IL-1β and TNF-α in serum samples from STZ-treated mice was greatly abated in STZ + shOGT group (Fig. 6D-E). ACTA2 and LMOD1 protein levels were down-regulated but PCNA protein expression was up-regulated in STZ mice, whereas these protein changes were obviously abrogated after OGT knockdown in STZ mice (Fig. 6F), indicating that shOGT inhibited vascular dedifferentiation in STZ mice. OGT downregulation could abate kidney injury and serum inflammation, and OGT positively regulated MAGI1 level in STZ-induced DM mice.

## Discussion

Vasculopathy is crucial to the occurrence and progression of DM-associated vascular complications. Exploration of the molecular mechanism of VSMC dysfunction under the condition of DM is of great significance. Currently, OGT/MAGI1 was found to participate in the processes of inflammation and dedifferentiation in HG-constructed VSMCs.

In consistent with the previous study, the upregulation of MAGI1 was also found in DM patients currently. Additionally, HG induced the level elevation of MAGI1 in VSMCs. Abnormal expression of MAGI1 implicated its potential function in diabetic vasculopathy. MAGI1 has exhibited pivotal implication in cancer and vascular biology [19]. For example, MAGI1 is involved in activating endothelial nitric oxide synthase and enhancing nitric oxide production in endothelial cells [20]. Also, MAGI1 was confirmed to drive atherosclerosis via affecting endothelial activation and endoplasmic reticulum stress [21]. However, the role of MAGI1 in vascular dysfunction resulting from DM is not clear. The function of MAGI1 depends on PDZ scaffolds that bind to target protein to further regulate signaling pathway, not only in stabilizing intercellular contacts and inhibiting invasiveness of endothelial cells [22], but also in regulating PTEN-mediated PI3K/AKT pathway in vascular functions [19]. In addition, MAGI1 can interact with over 110 types of cellular proteins [23], indicating the scaffold function of MAGI1 is cell universal. Therefore, exploring the role of MAGI1 in VSMCs can expand the understanding of cell functional spectrum of MAGI1.

As the principal constituent of the medial layer in arteries, VSMCs are indispensable for maintaining the good performance of the vasculature. VSMCs show the enhanced abilities of proliferation and migration in the formation of atherosclerotic plaque caused by DM [24]. VSMCs are correlated with the dedifferentiation exhibiting the phenotypic conversion from a quiescent “contractile” state to a highly migratory and proliferative “synthetic” phenotype in response to vascular injury and repair [25]. The current results demonstrated that HG led to cell hyperproliferation, migration and invasion, but MAGI1 knockdown suppressed HG-induced these vascular changes in VSMCs. Furthermore, the genes associated with VSMC differentiation were measured. ACTA2 gene can encode α-smooth muscle actin that is conductive to vascular contraction [26], and LMOD1 also acts as a VSMC differentiation marker [18]. Through the expression change of ACTA2 and LMOD1, it was found that HG inhibited VSMC differentiation and MAGI1 downregulation restored this effect to enhance VSMC differentiation and reduce VSMC dedifferentiation. Inflammation is an important feature in vascular diseases including DM [27], and it is considered as one of the inducing factor for VSMC senescence [28]. The detection for inflammatory cytokines demonstrated the inhibitory regulation of silencing MAGI1 in HG-induced inflammation in VSMCs. Moreover, MAGI1 overexpression exerted the opposite regulation of these cellular processes with regard to its downregulation in VSMCs. Therefore, this study concluded that MAGI1 expedited HG-induced vascular injury in VSMCs.

GlcNAcylation is important to the development of various diseases [29]. Dong et al.. reported that OGT-induced O-GlcNAcylation repressed the activation of astrocytes and inflammation through NF-κB signaling pathway [30]. By increasing O-GlcNAcylated SNAP-29 level, OGT could reduce cisplatin resistance in ovarian cancer [31]. The protein O-GlcNAcylation has been identified to participate in osteogenic differentiation and calcification of VSMCs in DM [32]. Herein, OGT was shown to promote O-GlcNAc level of MAGI1. In terms of VSMC function, HG-induced VSMC injury was mitigated by knockdown of OGT via downregulating MAGI1. All in all, OGT enhanced vascular injury in DM through elevating MAGI1 expression by O-GlcNAcylation. In STZ-induced DM model in mice, OGT was up-regulated and it could enhance the expression of MAGI1. STZ-induced inflammation in serum samples from mice was alleviated by silence of OGT. In addition, DM is at higher risk of impairment of kidney function. The kidney become larger and the glomerular filtration rate is high in the onset of DM [33]. After induction of STZ, kidney injury was observed in mice and the expression reduction of OGT prevented from it. Thus, OGT was considered to promote the development of DM in mice.

PI3K/AKT signaling pathway is an important downstream pathway after disruption of oxidant-antioxidant balance in the development of diabetic vascular complications [34]. MAGI1 can bind to PTEN (a regulatory factor of PI3K) through PDZ domains [35]. Herein, MAGI1 was shown to activate the PI3K/AKT pathway in HG-induced VSMCs. O-GlcNAcylation has revealed the cross-regulation of PI3K/AKT/mTOR signaling pathway in human chronic diseases, including DM [36]. According to the collected results, OGT also affected the PI3K/AKT pathway through upregulating MAGI1. Thus, OGT/MAGI1 was implicated in the regulation of VSMC disorder via mediating the PI3K/AKT pathway, revealing a potential OGT/MAGI1/PI3K-AKT novel regulatory module. In addition, its role in diabetic VSMC dysfunction likely intersects with other established pathways. For instance, the PI3K/AKT signaling is known to enhance oxidative stress and promote NF-κB-driven inflammation [37], both central to diabetic vasculopathy [38, 39]. Future studies should aim to map the precise crosstalk and potential feed-forward loops between this axis and other key pathways, which may together form an integrated signaling network driving VSMC pathology.

The clinical translation of our findings positions the OGT/MAGI1 axis as a promising therapeutic target with distinct advantages over conventional approaches for diabetic vascular complications. Current management strategies primarily address systemic risk factors through glycemic control and lipid management, yet often fail to halt the progression of underlying vascular pathology. Our work demonstrates that targeted disruption of the OGT-MAGI1 interaction specifically mitigates VSMC dysfunction—a core pathological process in diabetic vasculopathy—while potentially preserving physiological PI3K/AKT signaling in other cell types. This cellular specificity represents a significant advantage over broad-spectrum approaches. Although further validation in advanced disease models is warranted, pharmacological targeting of this axis could provide a mechanistically grounded strategy for patients with established vascular complications, complementing rather than replacing existing standard of care. The development of tissue-specific modulators of OGT/MAGI1 signaling may thus fill a critical gap in our current therapeutic arsenal against diabetic vascular disease. While this study has primarily focused on the role of the OGT/MAGI1 axis in VSMC dysfunction, it is plausible that this signaling pathway may also contribute to other diabetic vascular pathologies. For instance, in diabetic retinopathy, sustained hyperglycemia and O-GlcNAcylation alterations are known to disrupt the blood-retinal barrier. Given MAGI1’s established function in maintaining endothelial and pericyte junctional integrity, it is conceivable that hyperglycemia-induced OGT activation and subsequent MAGI1 O-GlcNAcylation could impair MAGI1’s scaffolding role at cell-cell contacts, thereby promoting vascular hyperpermeability—a hallmark of early retinopathy. Similarly, in diabetic nephropathy, podocyte injury and glomerular endothelial dysfunction are key events. The OGT/MAGI1 axis might influence the integrity of the glomerular filtration barrier, as MAGI1 is critically localized in podocyte foot processes and slit diaphragms. Dysregulation of this axis could potentially lead to podocyte detachment and proteinuria. Future studies are warranted to directly investigate the involvement and mechanistic details of OGT-mediated MAGI1 O-GlcNAcylation in these specific microvascular complications, which could unveil new therapeutic avenues for mitigating multi-organ damage in diabetes. Additionally, while targeting the OGT/MAGI1 axis presents a novel therapeutic strategy, significant challenges remain, particularly regarding the risk of disrupting essential physiological O-GlcNAcylation in other tissues with systemic inhibition. Future research must focus on developing tissue- or cell-selective delivery approaches to mitigate potential off-target effects and fully evaluate the safety profile of this strategy.

However, there are still some limitations. Firstly, while the present study did not systematically include osmotic controls (e.g., mannitol) in all experiments, this omission is mitigated by our observation that osmotic pressure had no significant effect on O-GlcNAc protein expression and the PI3K/AKT signaling pathway (Supplementary Fig. 2A and 2B). The mannitol control can better contribute to improving the experimental design. Thus, a mannitol control will be included in the further study, to completely eliminate the interference from hypertonicity. Secondly, in animal experiments, kidney histology was analyzed because kidney is the core target organ of diabetic vascular complications, but classical vascular functional assays (e.g., aortic ring contraction and aortic plaque formation) are lacked. Due to the restrains of research stage and technology, these assays will be performed in the subsequent plan. Additionally, this study has not explored the correlation of MAGI1 level with diabetic vascular complications in clinical studies. Future study will collect renal puncture tissues from patients with diabetic nephropathy and lower extremity arterial surgery specimens from patients with diabetic peripheral vascular disease, and analyze the levels of MAGI1, the modification status of O-GlcNAcylation, and the association between MAGI1 and the severity of clinical complications (e.g., glomerular filtration rate, urine protein quantification, and the degree of vascular stenosis). Moreover, while the current study demonstrates that the OGT/MAGI1 axis promotes key cellular processes in VSMCs under diabetic conditions and correlates its activity with serum markers of differentiation and renal collagen deposition, we acknowledge its limitations in directly assessing vascular functional and structural endpoints. Specifically, the absence of direct hemodynamic measurements (e.g., blood pressure, aortic pulse wave velocity), quantitative analyses of atherosclerotic burden (e.g., aortic plaque area, intima-media thickness), and in situ visualization of VSMC phenotypic switching within the vasculature (e.g., via co-immunofluorescence for PCNA and α-SMA) means that the link between the OGT/MAGI1 axis and macrovascular dysfunction, a critical aspect of diabetic complications, remains partially indirect. Future studies will therefore prioritize these direct vascular assessments to more conclusively establish the pathophysiological role of this signaling pathway in diabetic vasculopathy and to solidify its relevance as a therapeutic target for vascular complications in diabetes. Finally, while this study identified elevated circulating MAGI1 protein in diabetic patients, it remains to be determined whether serum MAGI1 levels directly reflect its expression within the vascular wall. Future studies measuring MAGI1 in vascular tissues alongside serum samples are warranted to establish a more direct correlation and to validate serum MAGI1 as a specific biomarker for vascular pathophysiology in DM.

**Fig. 7:**
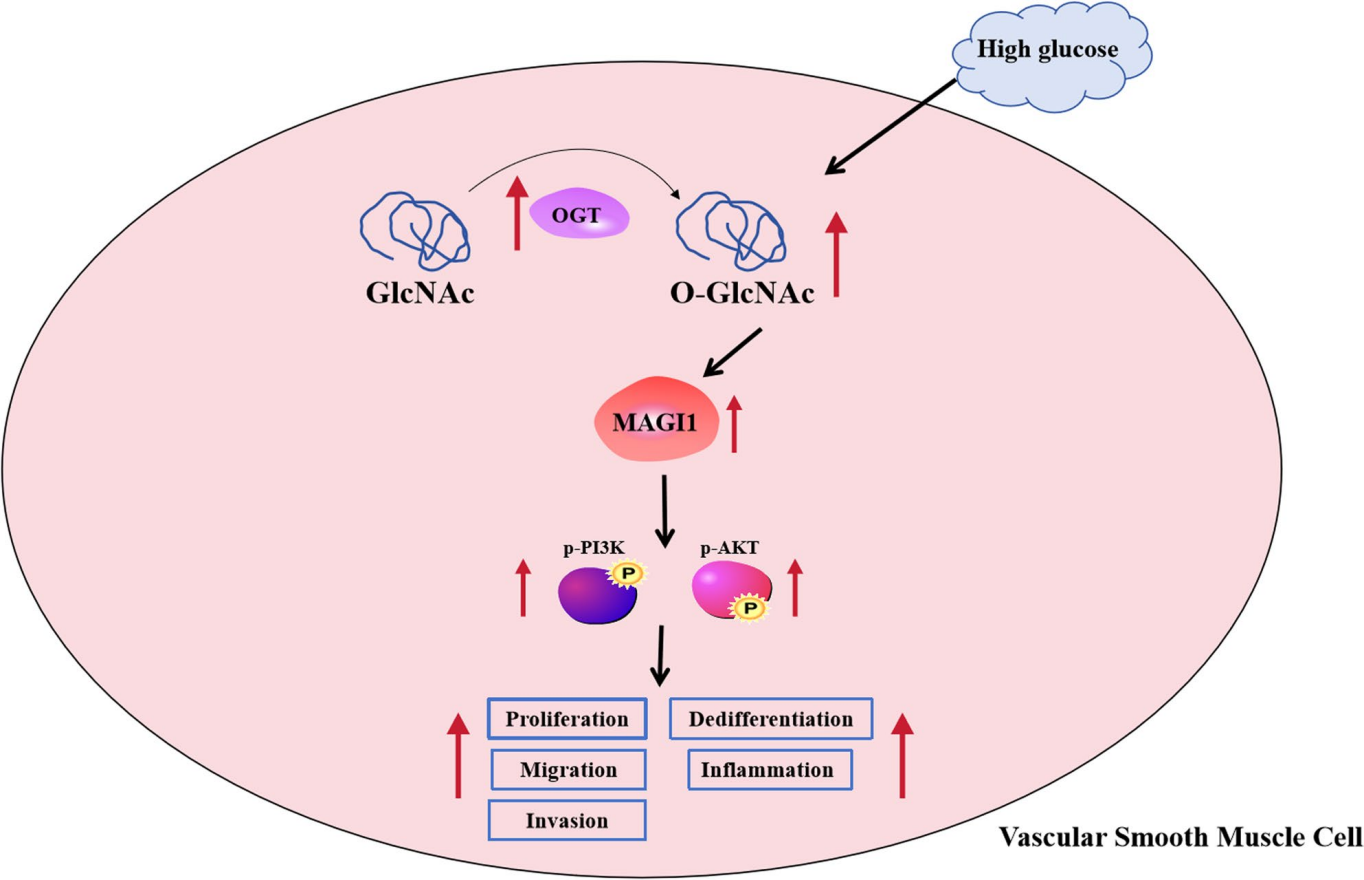
OGT increased the O-GlcNAc level of MAGI1 to promote proliferation, migration, invasion, inflammation and dedifferentiation of HG-treated VSMCs via activating the PI3K/AKT pathway

In conclusion, these findings for the first time show that OGT up-regulates MAGI1 by promoting MAGI1 O-GlcNAcylation, thereby accelerating VSMC proliferation, migration, invasion, inflammation, and dedifferentiation under HG conditions (Fig. 7). The OGT/MAGI1 axis significantly facilitated vascular dysfunction in the progression process of DM.

## Supplementary Information

Supplementary Material 1. Supplementary Figure 1: The effect of site-directed mutation on O-GlcNac level and cell function. (A) O-GlcNac protein was detected in VSMCs treated with HG, HG+MAGI1-WT, HG+MAGI1-S991A, HG+MAGI1-S1362A, and HG+MAGI1-DM. (B-D) VSMCs treated with HG, HG+MAGI1-WT, HG+MAGI1-S991A, HG+MAGI1-S1362A, and HG+MAGI1-DM were checked for cell viability (B), migration (C), and the protein levels of PI3K, p-PI3K, AKT, and p-AKT (D). *P < 0.05, **P < 0.01, ***P < 0.001.

Supplementary Material 2. Supplementary Figure 2: The effects of the osmo control on O-GlcNac level and the PI3K/AKT pathway. (A and B) VSMCs were treated with NG, Osmo, and HG, followed by evaluation of MAGI1 O-GlcNac level (A) and the protein levels of PI3K, p-PI3K, AKT, and p-AKT (B). **P < 0.01, ***P < 0.001, ns: non-significant.

Supplementary Material 3. Supplementary Figure 3: The reverse effects of overexpressing OGT in shOGT-transfected VSMCs. (A-D) VSMCs treated with HG, HG+shOGT, HG+shOGT+OGT-WT, and HG+shOGT+OGT-H558A, followed by detection of OGT level (A), MAGI1 O-GlcNac level (B), cell viability (C), and cell migration (D). *P < 0.05, **P < 0.01, ***P < 0.001, ns: non-significant.

Supplementary Material 4.

Supplementary Material 5.

Supplementary Material 6.

## Data Availability

The datasets used and analysed during the current study are available from the corresponding author on reasonable request.
