# Supplementary figures and images for: OGT-mediated O-GlcNAcylation of MAGI1 exacerbates high glucose-triggered inflammation and dedifferentiation of vascular smooth muscle cells by activating the PI3K/AKT pathway
Source: Hereditas. 2026 Jan 16;163:22. doi: 10.1186/s41065-026-00643-4 (PMC12892639; doi:10.1186/s41065-026-00643-4)

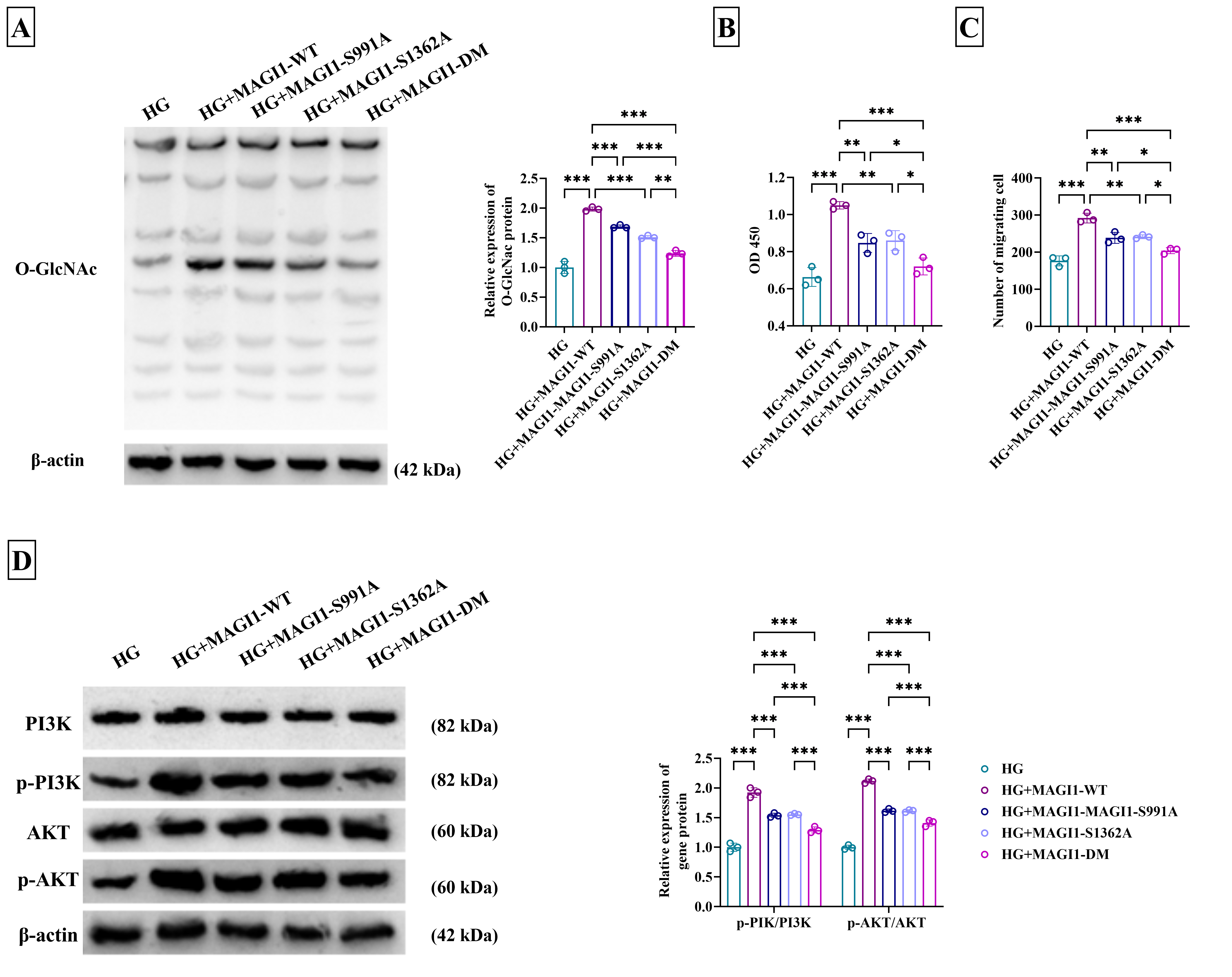

Supplement: Supplementary file 1 — Supplementary Material 1. Supplementary Figure 1: The effect of site-directed mutation on O-GlcNac level and cell function. (A) O-GlcNac protein was detected in VSMCs treated with HG, HG+MAGI1-WT, HG+MAGI1-S991A, HG+MAGI1-S1362A, and HG+MAGI1-DM. (B-D) VSMCs treated with HG, HG+MAGI1-WT, HG+MAGI1-S991A, HG+MAGI1-S1362A, and HG+MAGI1-DM were checked for cell viability (B), migration (C), and the protein levels of PI3K, p-PI3K, AKT, and p-AKT (D). *P < 0.05, **P < 0.01, ***P < 0.001. [file 41065_2026_643_MOESM1_ESM.tif]

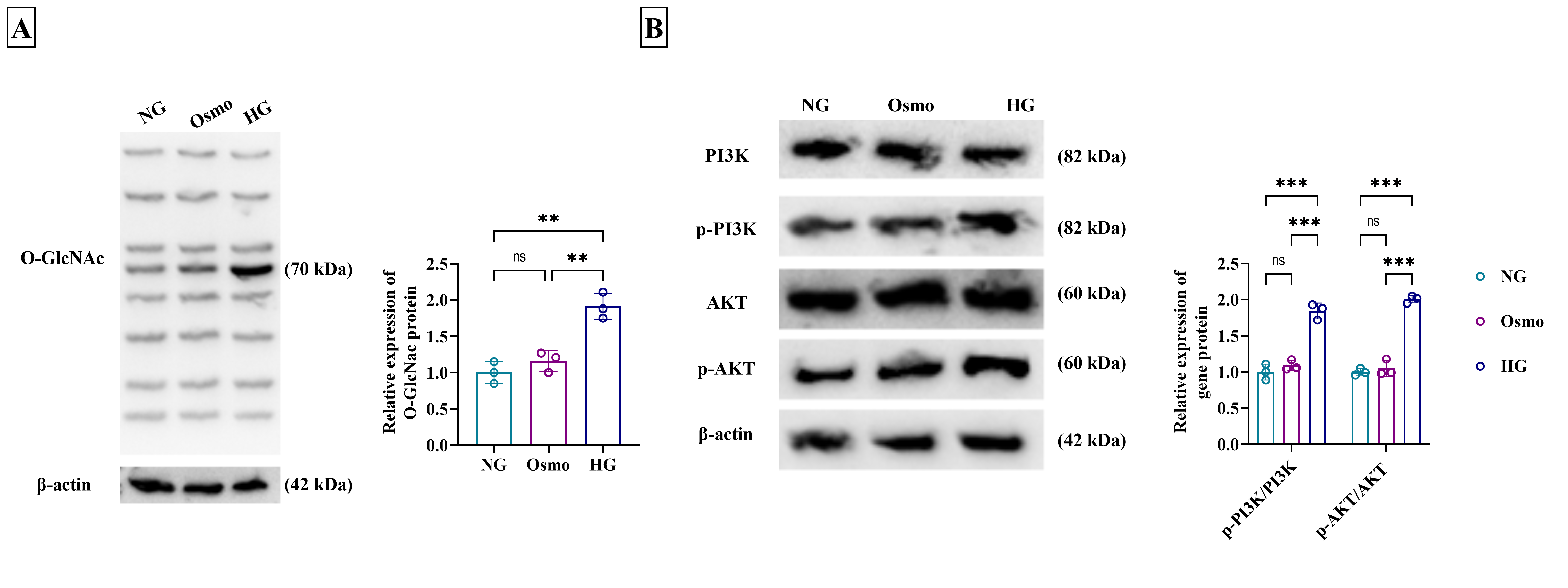

Supplement: Supplementary file 2 — Supplementary Material 2. Supplementary Figure 2: The effects of the osmo control on O-GlcNac level and the PI3K/AKT pathway. (A and B) VSMCs were treated with NG, Osmo, and HG, followed by evaluation of MAGI1 O-GlcNac level (A) and the protein levels of PI3K, p-PI3K, AKT, and p-AKT (B). **P < 0.01, ***P < 0.001, ns: non-significant. [file 41065_2026_643_MOESM2_ESM.tif]

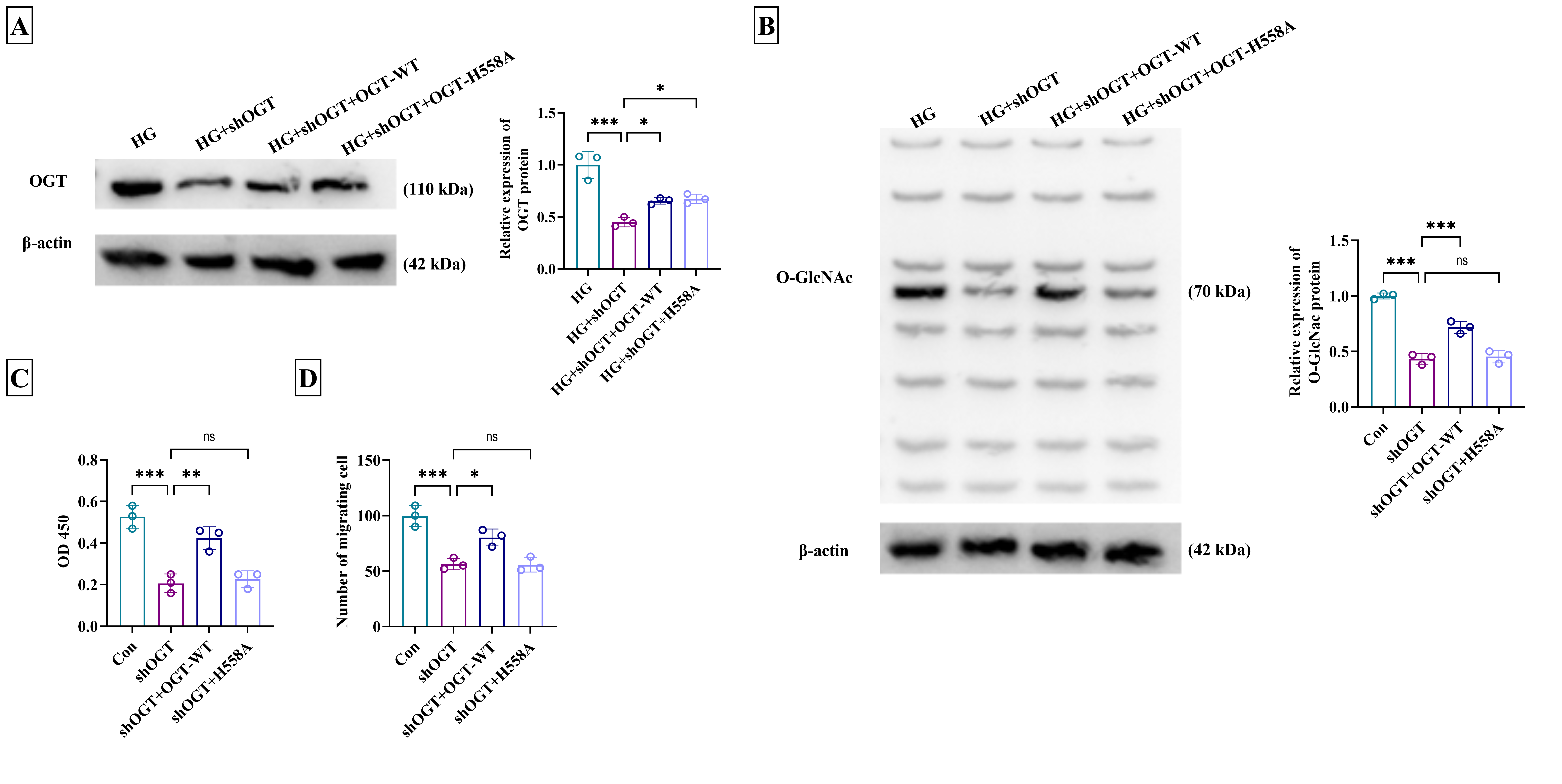

Supplement: Supplementary file 3 — Supplementary Material 3. Supplementary Figure 3: The reverse effects of overexpressing OGT in shOGT-transfected VSMCs. (A-D) VSMCs treated with HG, HG+shOGT, HG+shOGT+OGT-WT, and HG+shOGT+OGT-H558A, followed by detection of OGT level (A), MAGI1 O-GlcNac level (B), cell viability (C), and cell migration (D). *P < 0.05, **P < 0.01, ***P < 0.001, ns: non-significant. [file 41065_2026_643_MOESM3_ESM.tif]

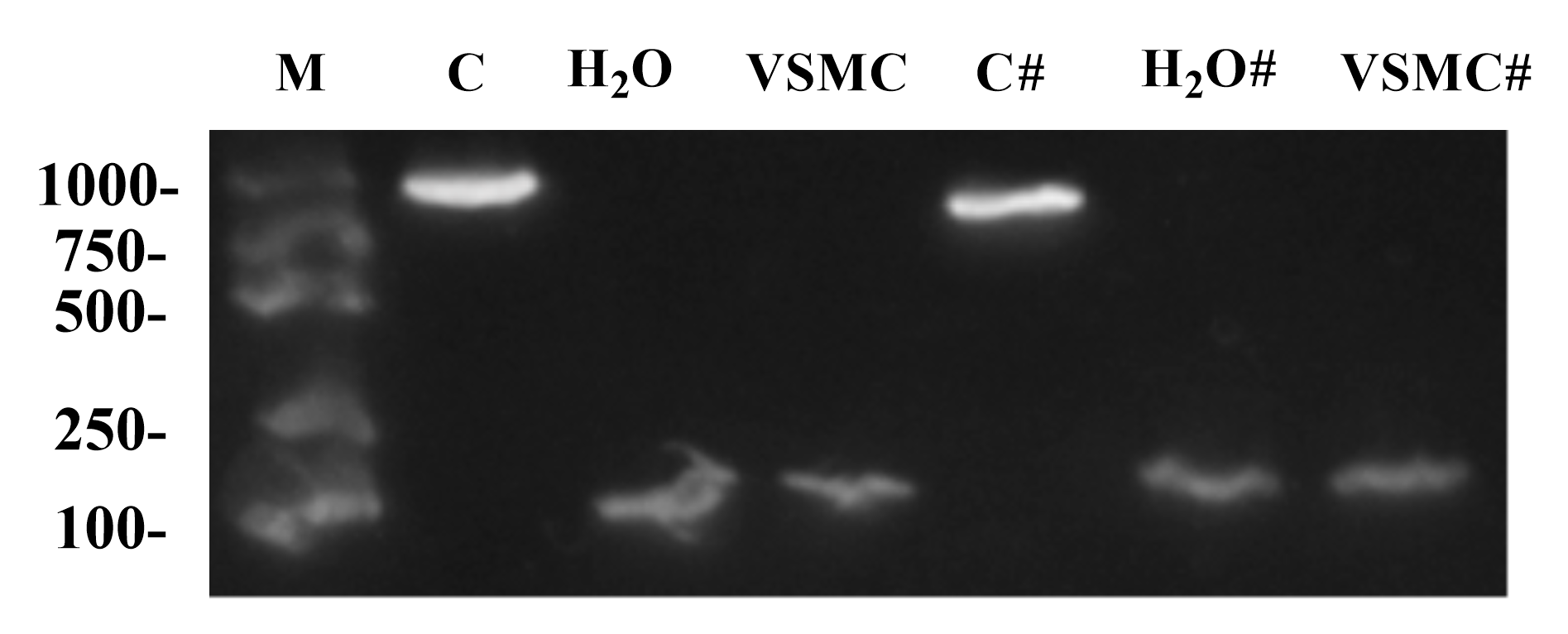

Supplement: Supplementary file 4 — Supplementary Material 4. [file 41065_2026_643_MOESM4_ESM.tif]
