# Supplementary material for: OGT-mediated O-GlcNAcylation of MAGI1 exacerbates high glucose-triggered inflammation and dedifferentiation of vascular smooth muscle cells by activating the PI3K/AKT pathway
Source: Hereditas. 2026 Jan 16;163:22. doi: 10.1186/s41065-026-00643-4 (PMC12892639; doi:10.1186/s41065-026-00643-4)
Supplement: Supplementary file 6 — Supplementary Material 6. [file 41065_2026_643_MOESM6_ESM.pdf]

Fig 1C

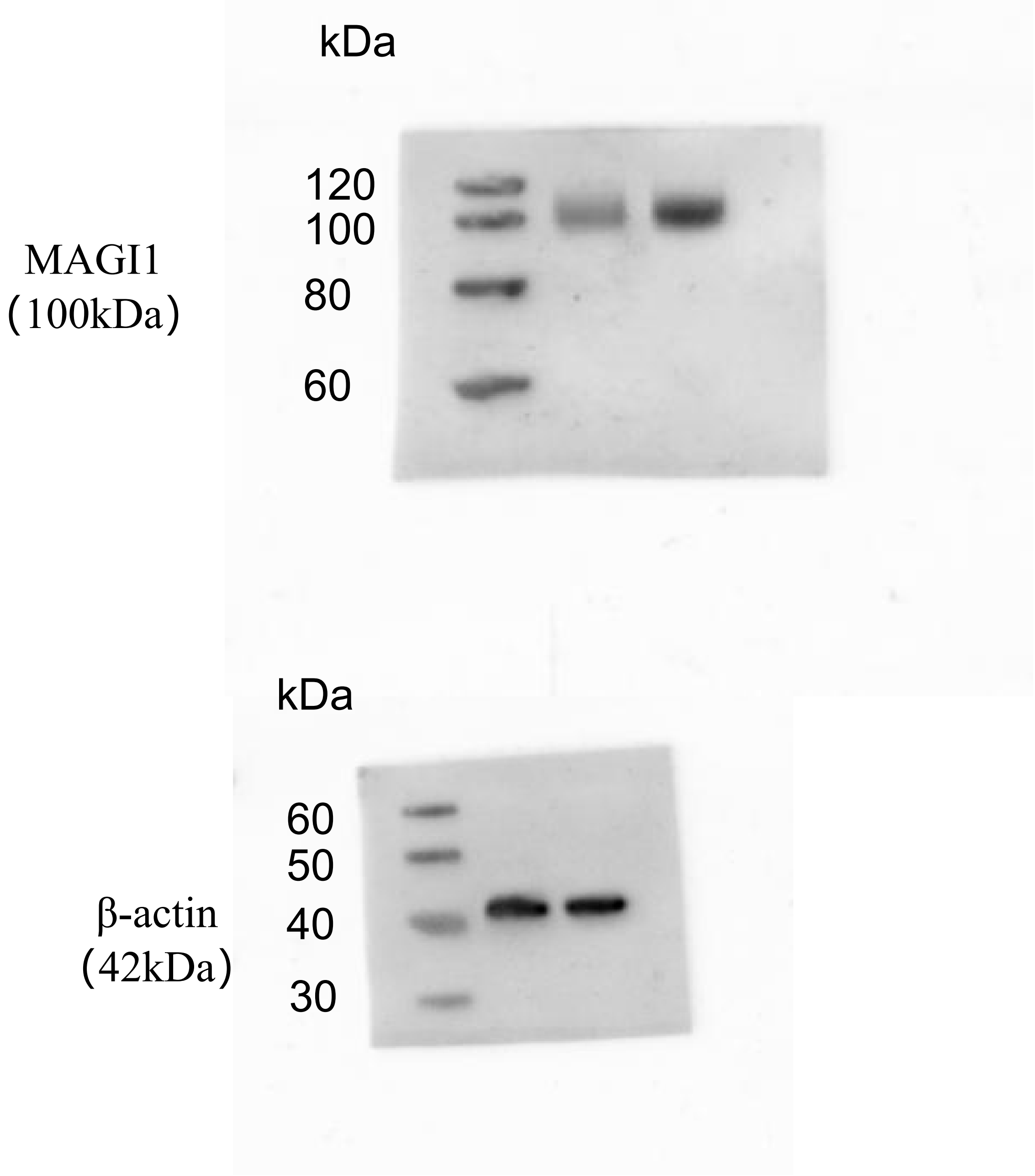

Fig1E

MAGI1
(100 kDa)

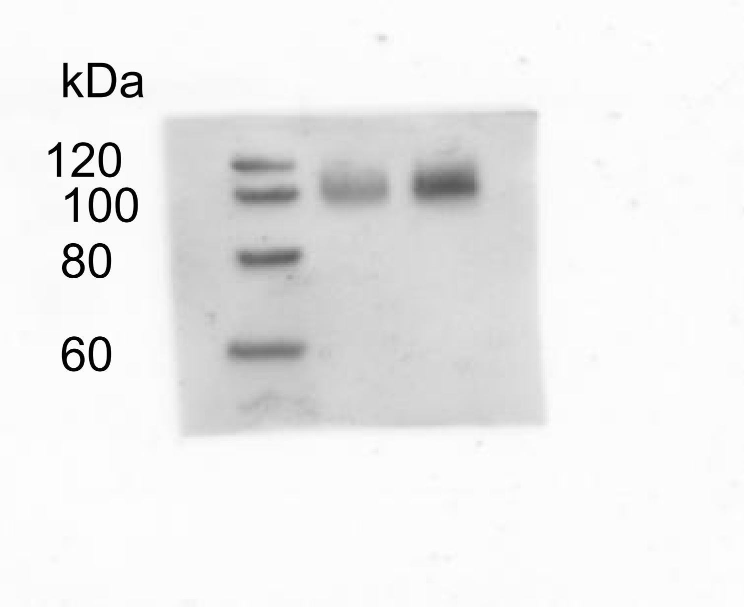

β-actin
(42 kDa)

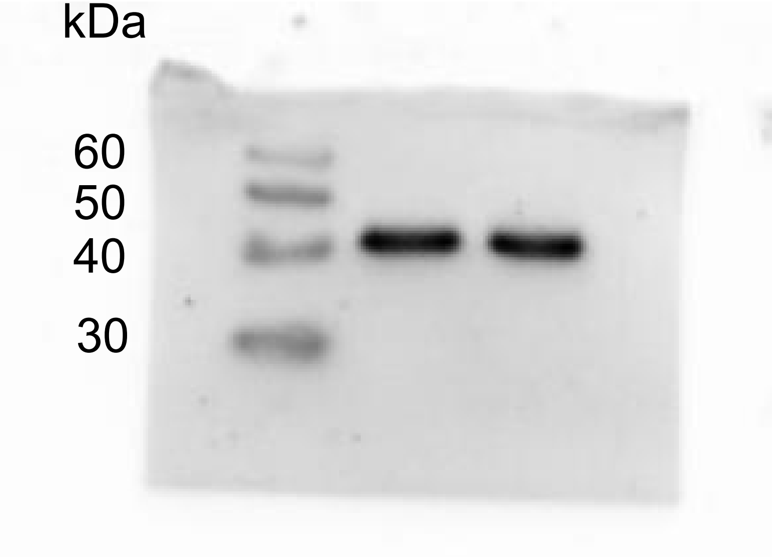

Fig2B

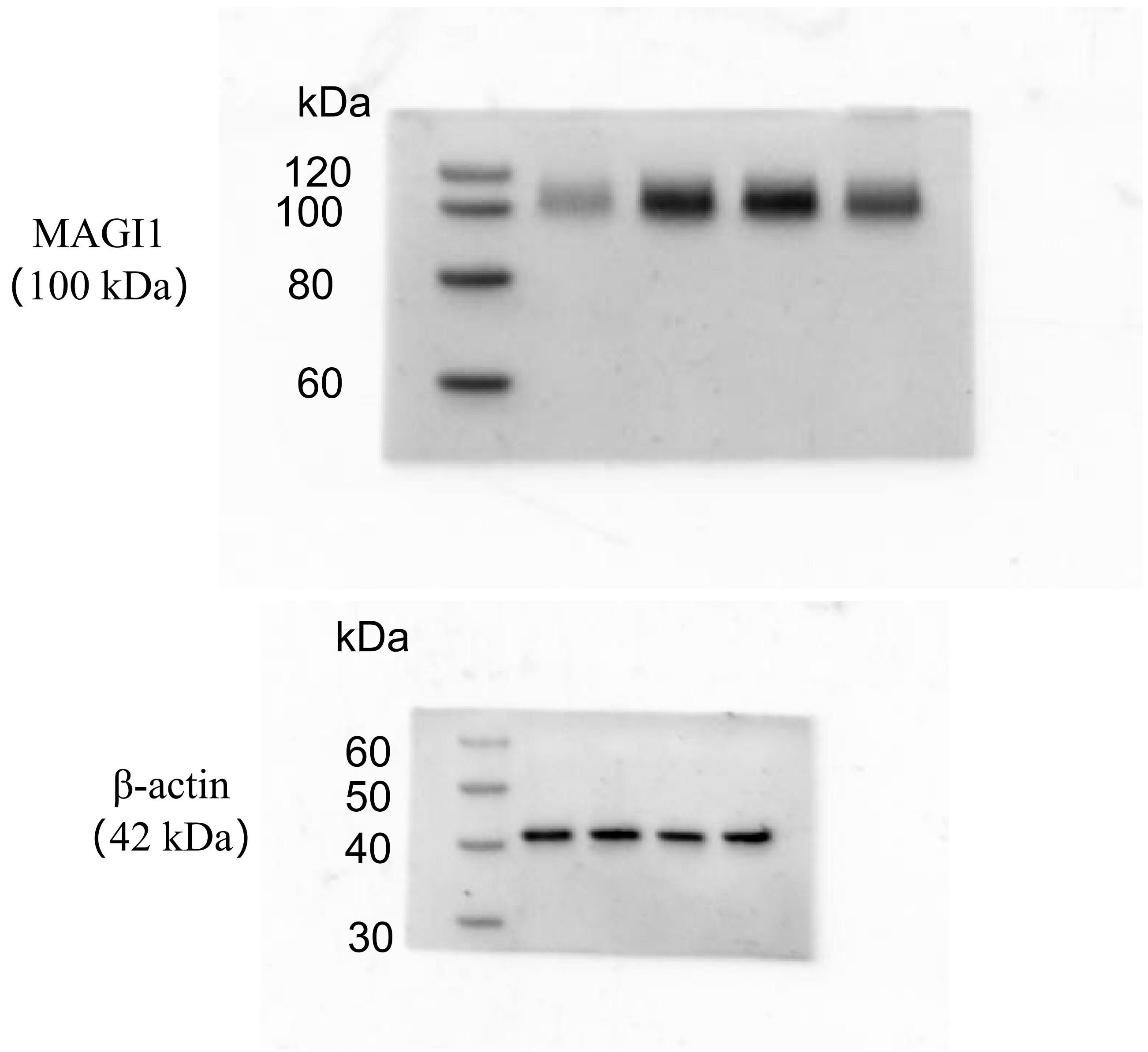

Fig 2I

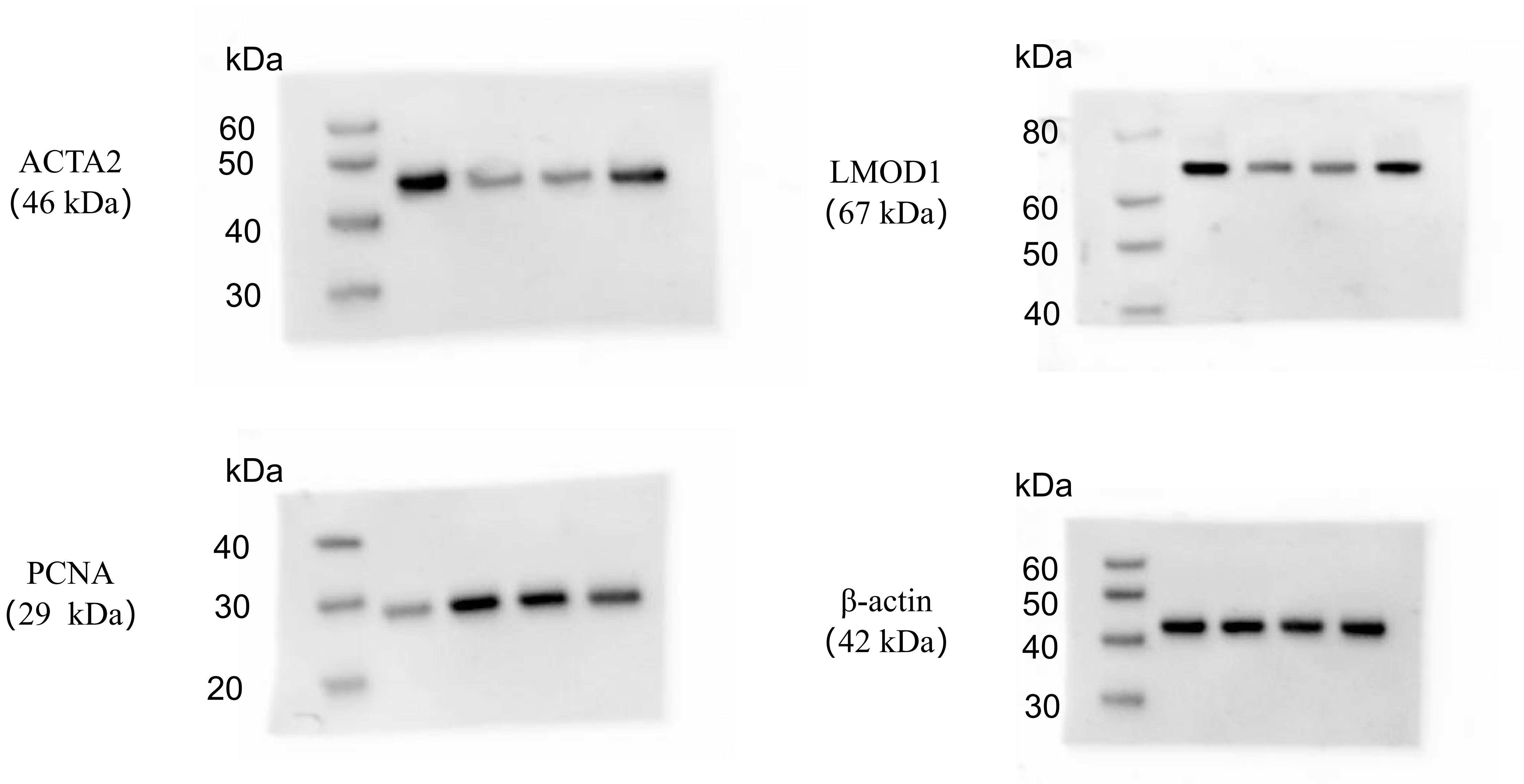

ACTA2
(46 kDa)
kDa
60
50
40
30
LMOD1
(67 kDa)
kDa
80
60
50
40
PCNA
(29 kDa)
kDa
40
30
20
β-actin
(42 kDa)
kDa
60
50
40
30

Fig 2J

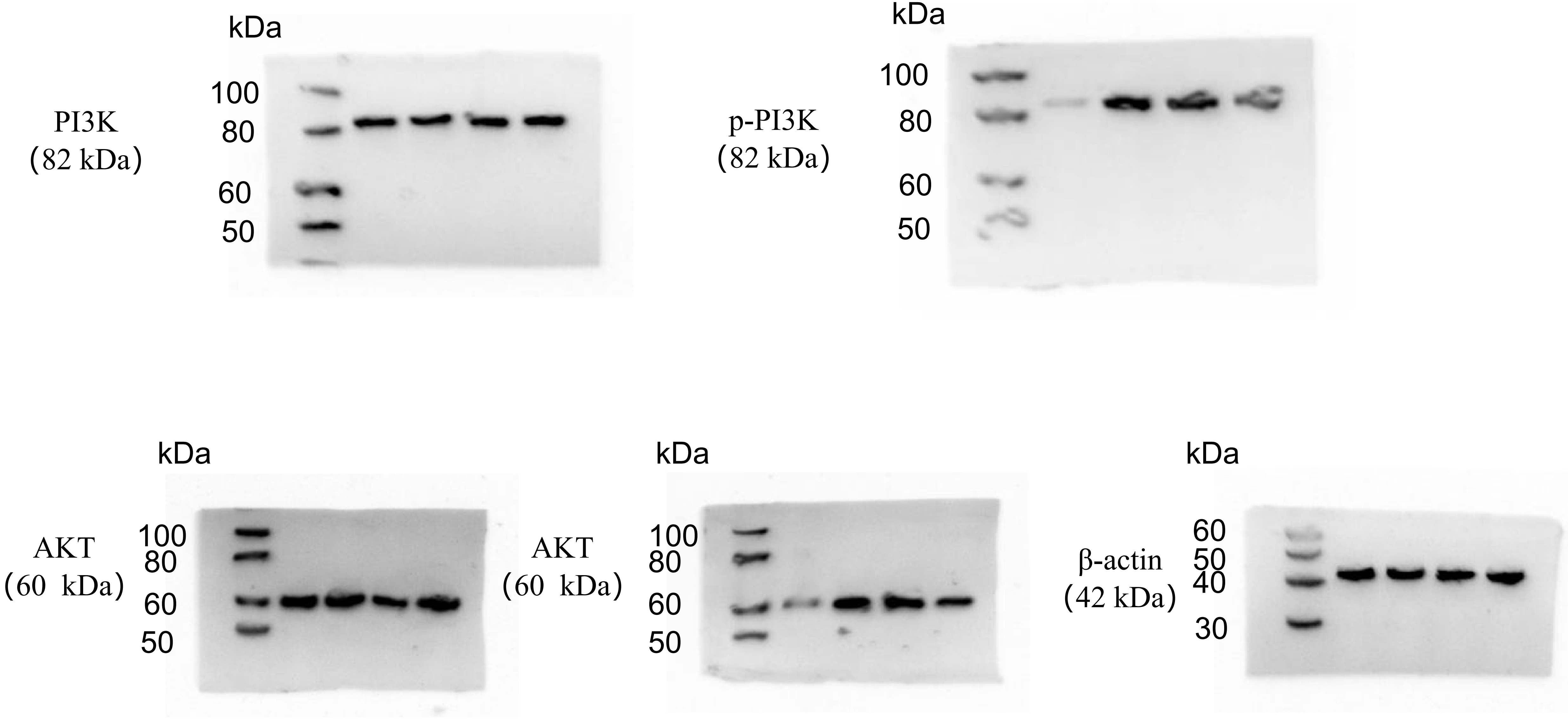
PI3K
(82 kDa)
kDa
100
80
60
50
p-PI3K
(82 kDa)
kDa
100
80
60
50
AKT
(60 kDa)
kDa
100
80
60
50
AKT
(60 kDa)
kDa
100
80
60
50
β-actin
(42 kDa)
kDa
60
50
40
30

Fig3B

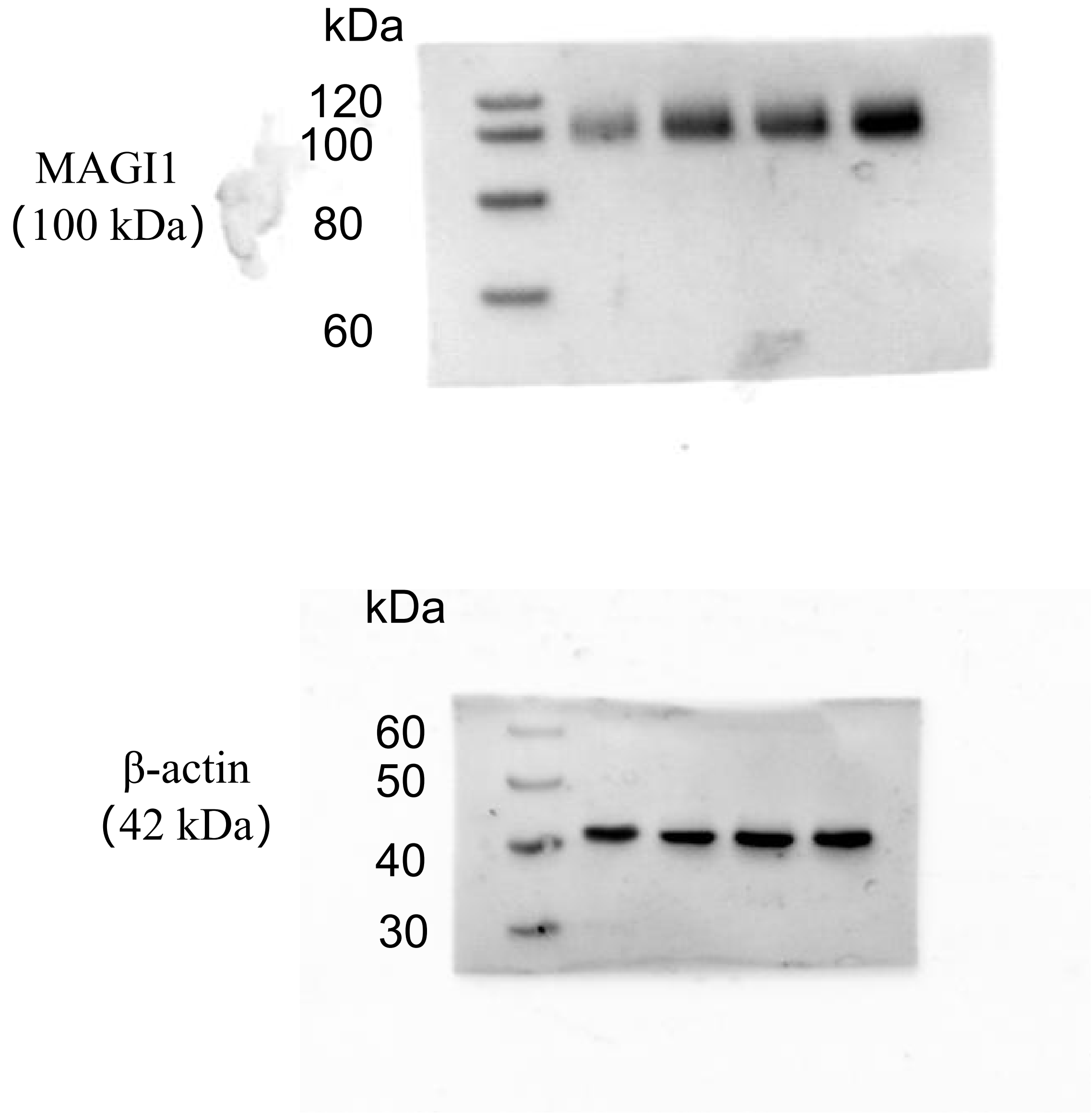

Fig 3I

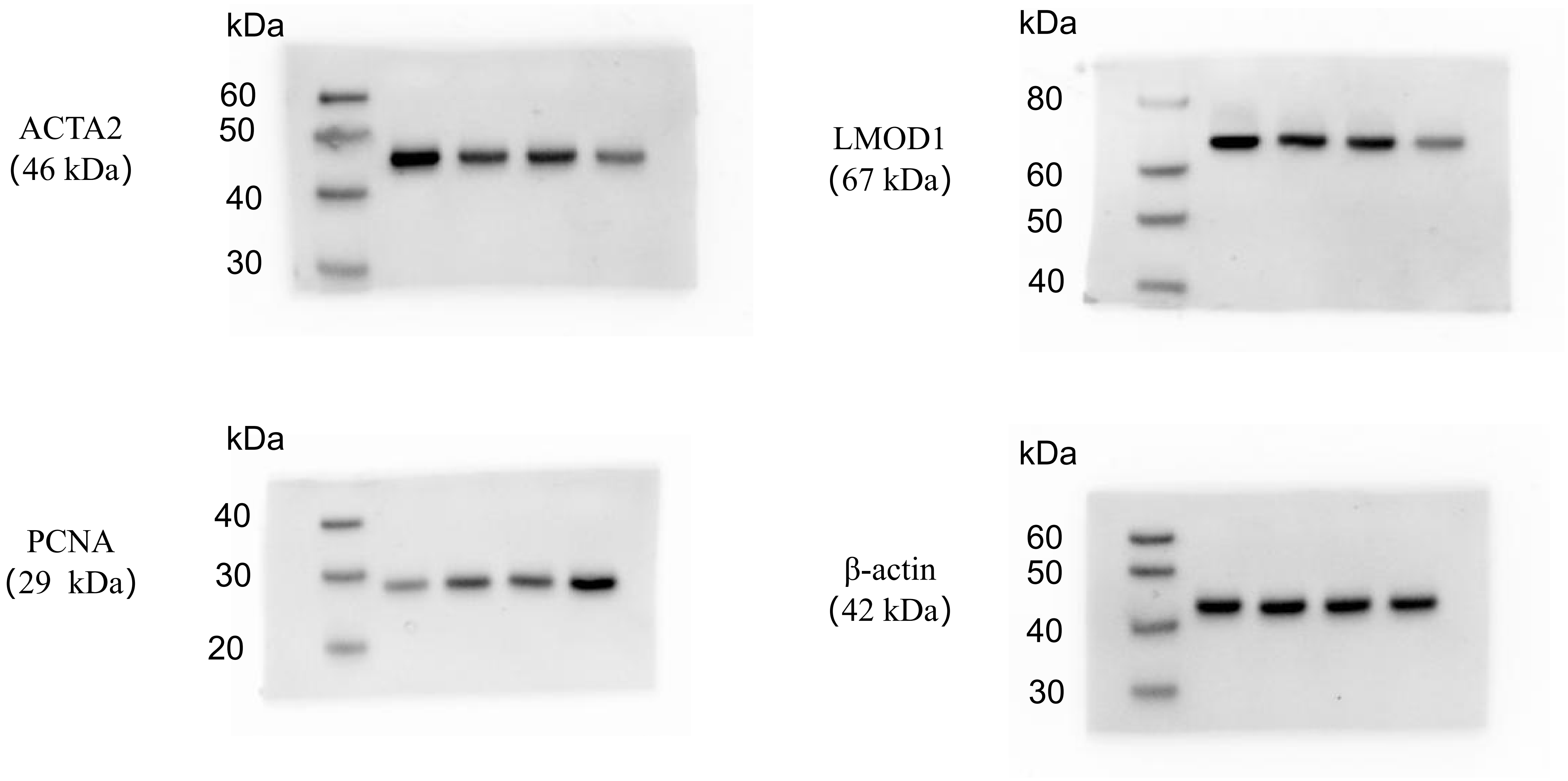
ACTA2
(46 kDa)
kDa
60
50
40
30
LMOD1
(67 kDa)
kDa
80
60
50
40
PCNA
(29 kDa)
kDa
40
30
20
β-actin
(42 kDa)
kDa
60
50
40
30

Fig 3J

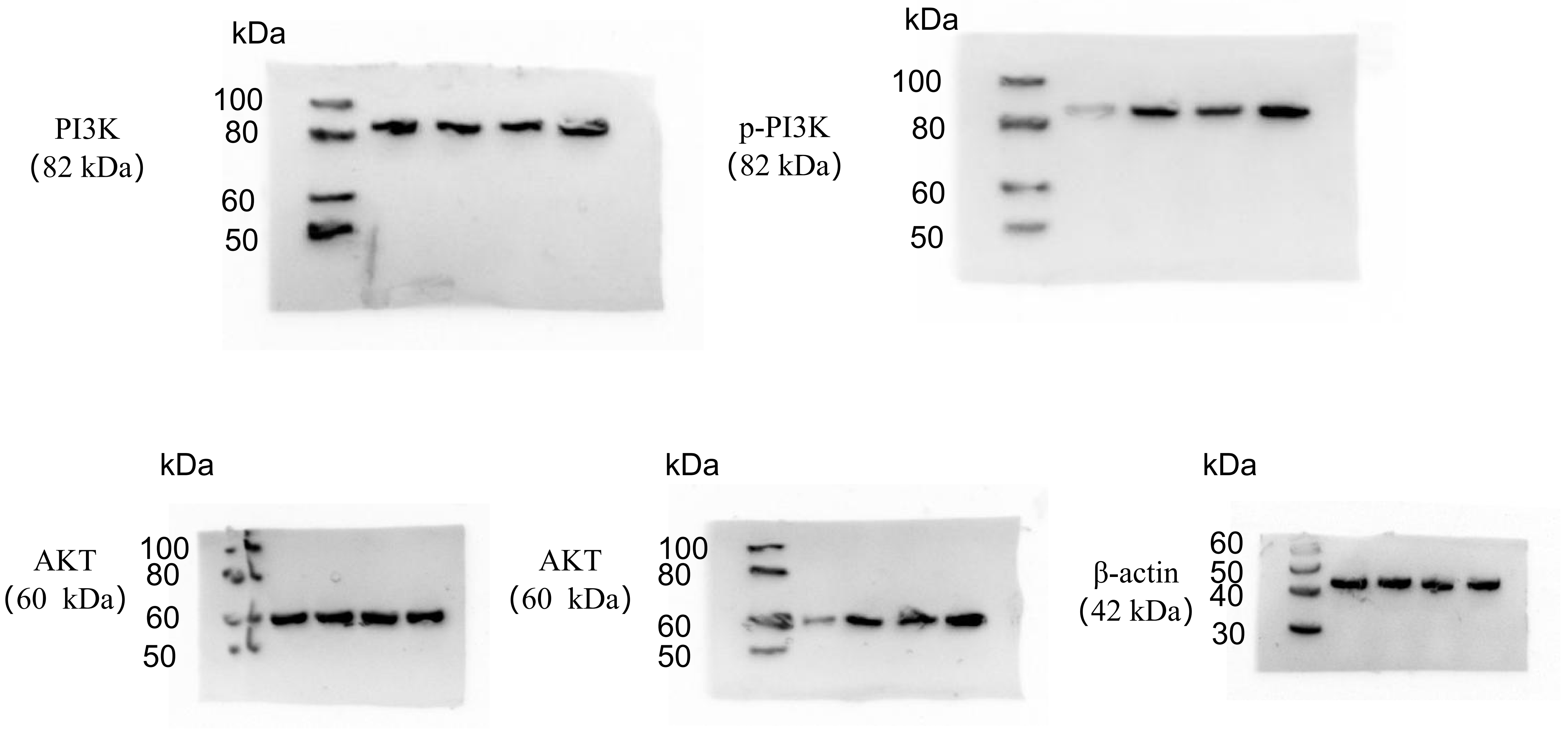

PI3K
(82 kDa)
kDa
100
80
60
50
p-PI3K
(82 kDa)
kDa
100
80
60
50
AKT
(60 kDa)
kDa
100
80
60
50
AKT
(60 kDa)
kDa
100
80
60
50
β-actin
(42 kDa)
kDa
60
50
40
30

Fig 4C

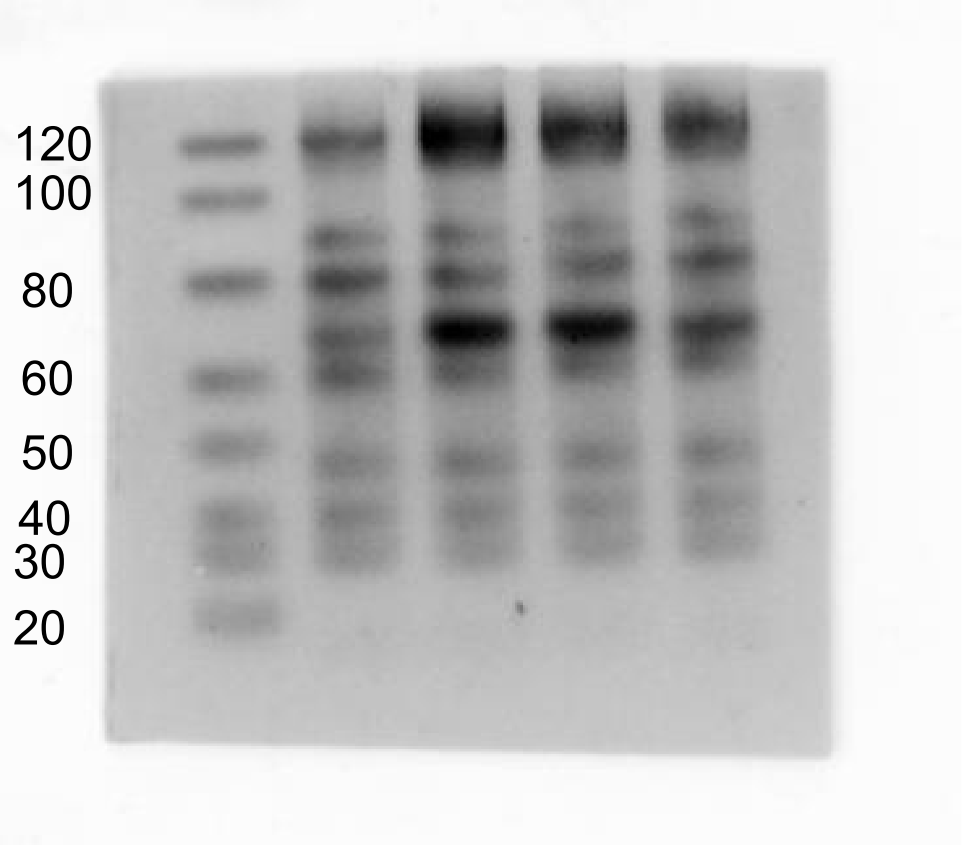

O-GlcNac
(70 kDa)
kDa
120
100
80
60
50
40
30
20

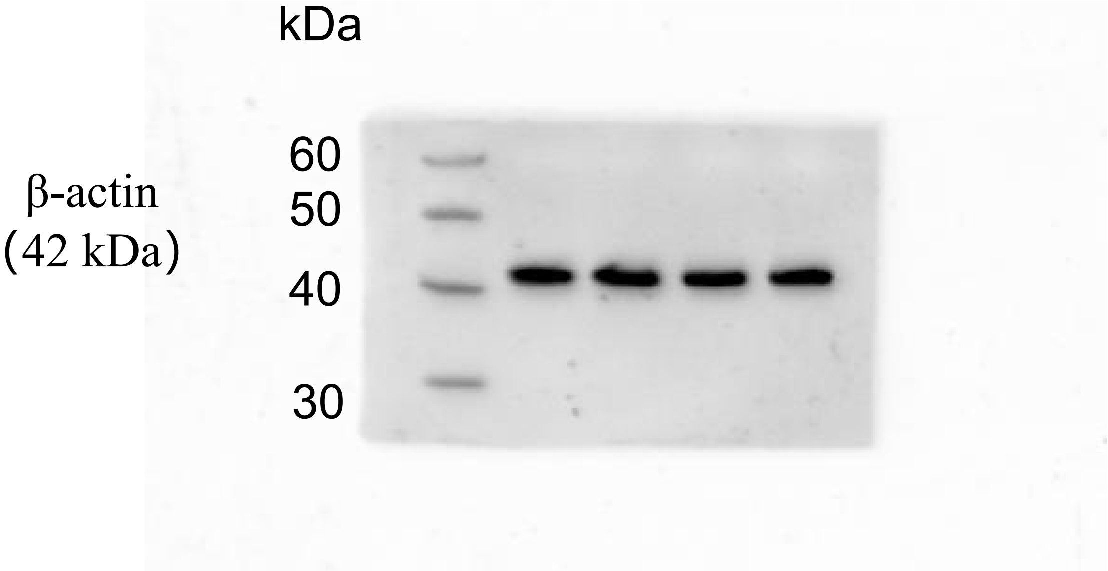

β-actin
(42 kDa)
kDa
60
50
40
30

Fig4D

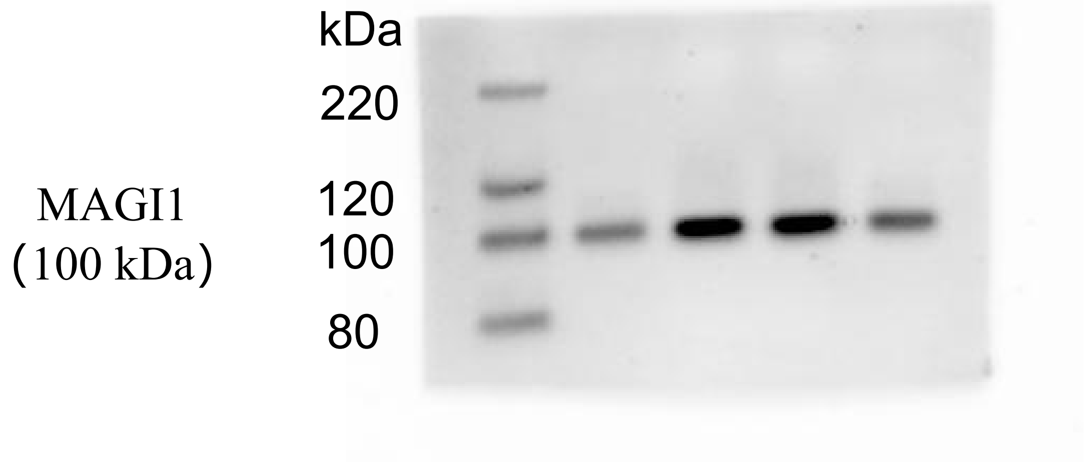
MAGI1
(100 kDa)
kDa
220
120
100
80

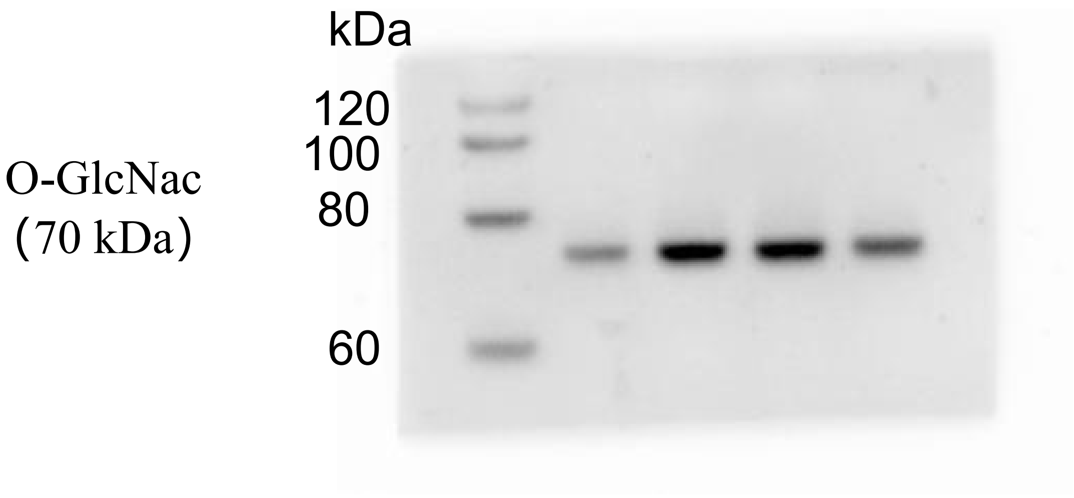
O-GlcNac
(70 kDa)
kDa
120
100
80
60

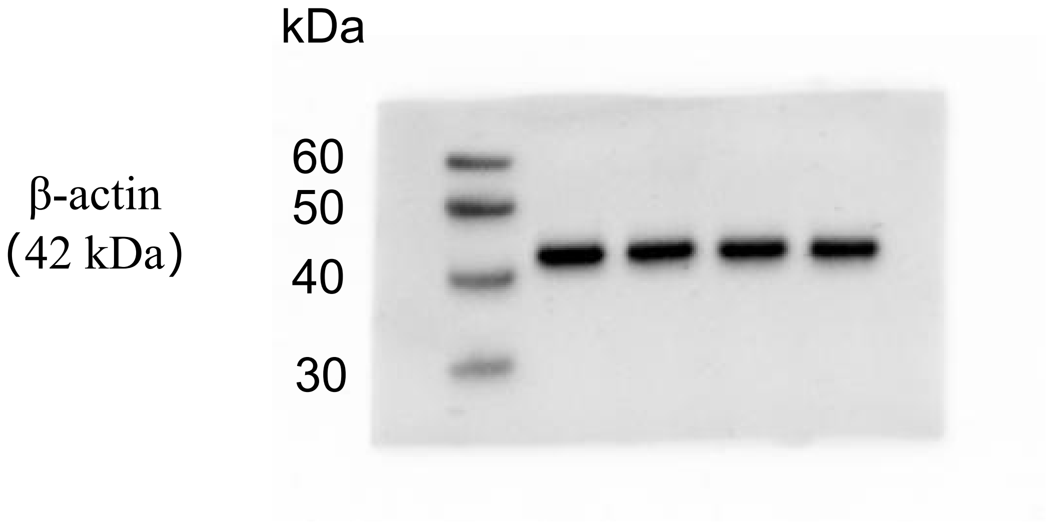
β-actin
(42 kDa)
kDa
60
50
40
30

Fig5A

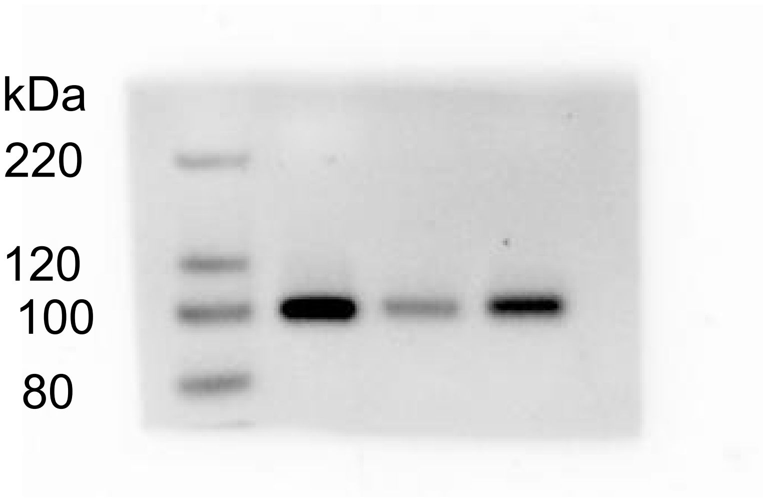

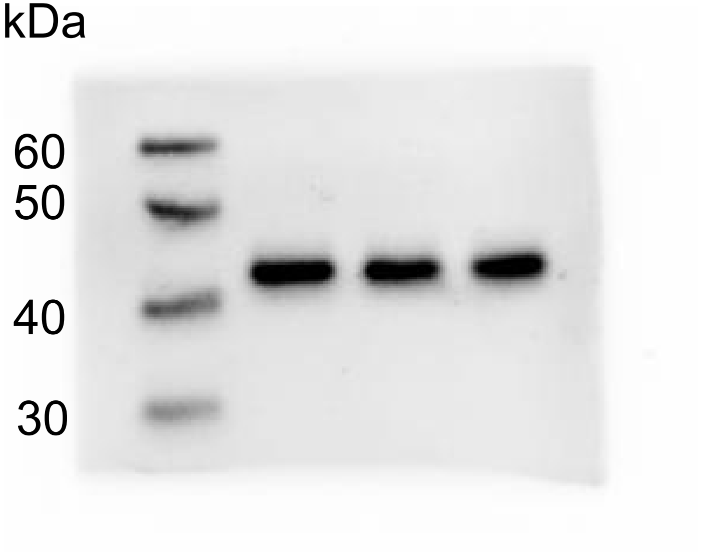

Fig 5I

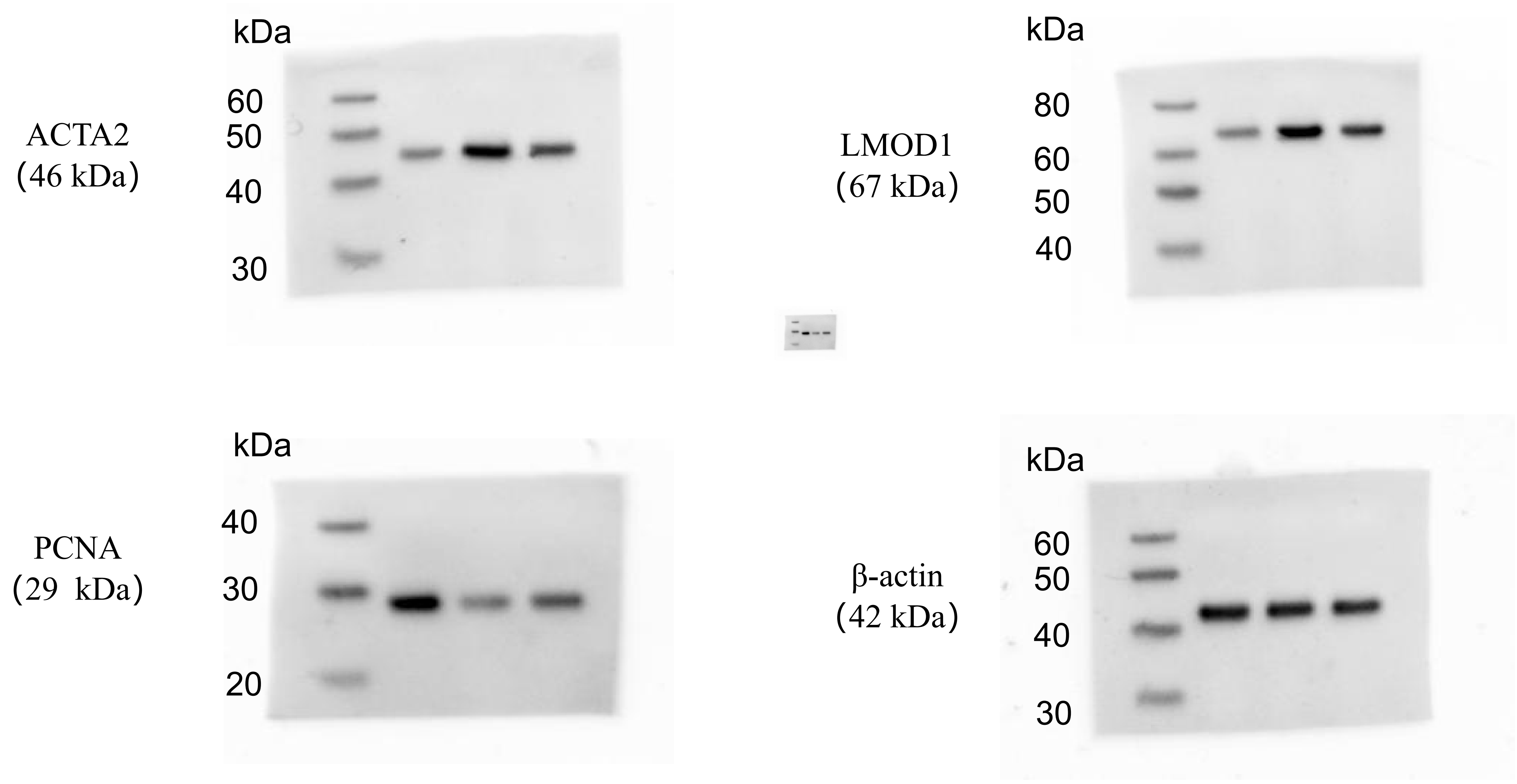
ACTA2
(46 kDa)
kDa
60
50
40
30
LMOD1
(67 kDa)
kDa
80
60
50
40
PCNA
(29 kDa)
kDa
40
30
20
β-actin
(42 kDa)
kDa
60
50
40
30

Fig 5J

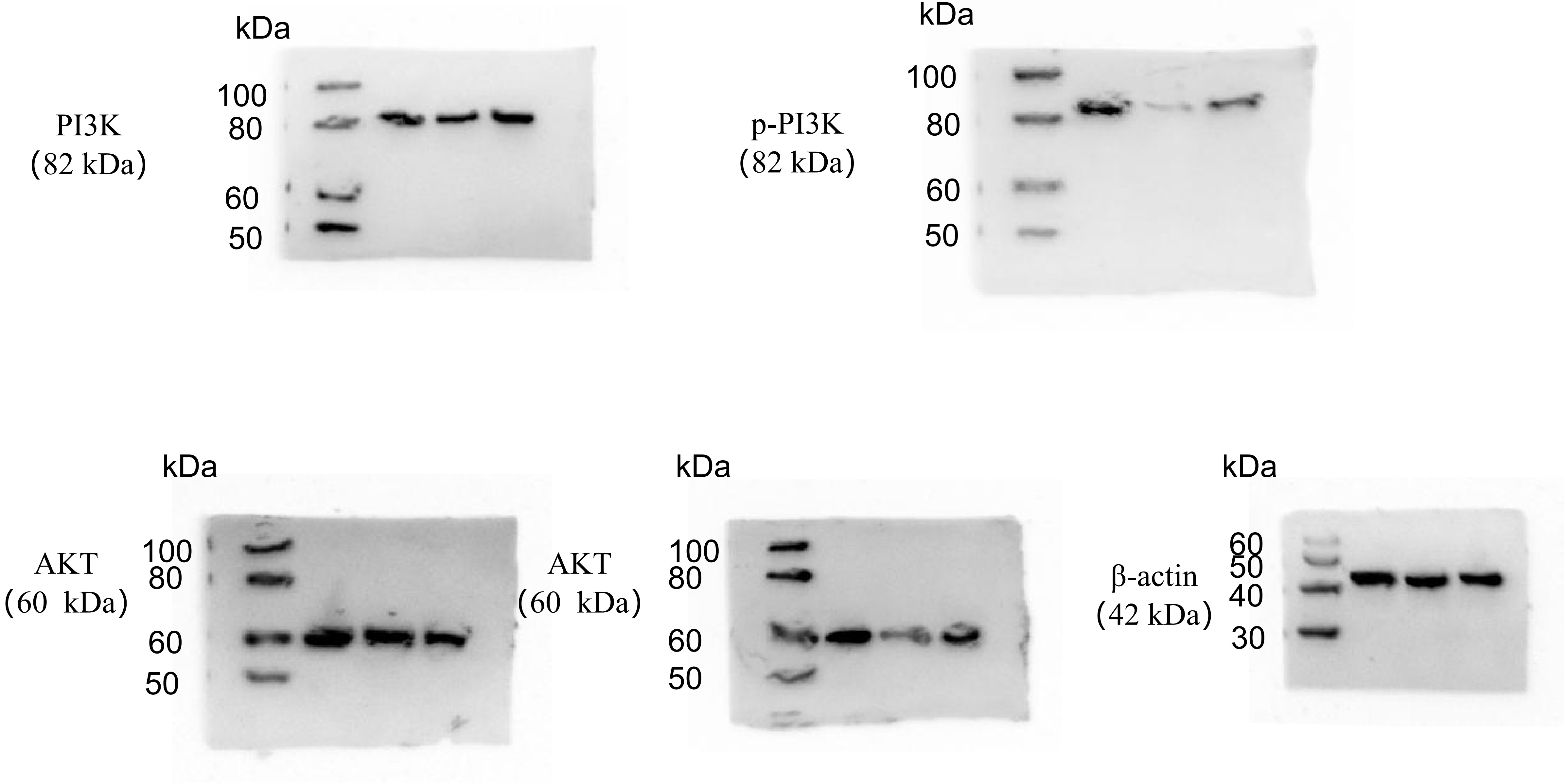

kDa
100
80
60
50
PI3K
(82 kDa)
kDa
100
80
60
50
p-PI3K
(82 kDa)
kDa
100
80
60
50
AKT
(60 kDa)
kDa
100
80
60
50
AKT
(60 kDa)
kDa
60
50
40
30
β-actin
(42 kDa)

Fig S1A

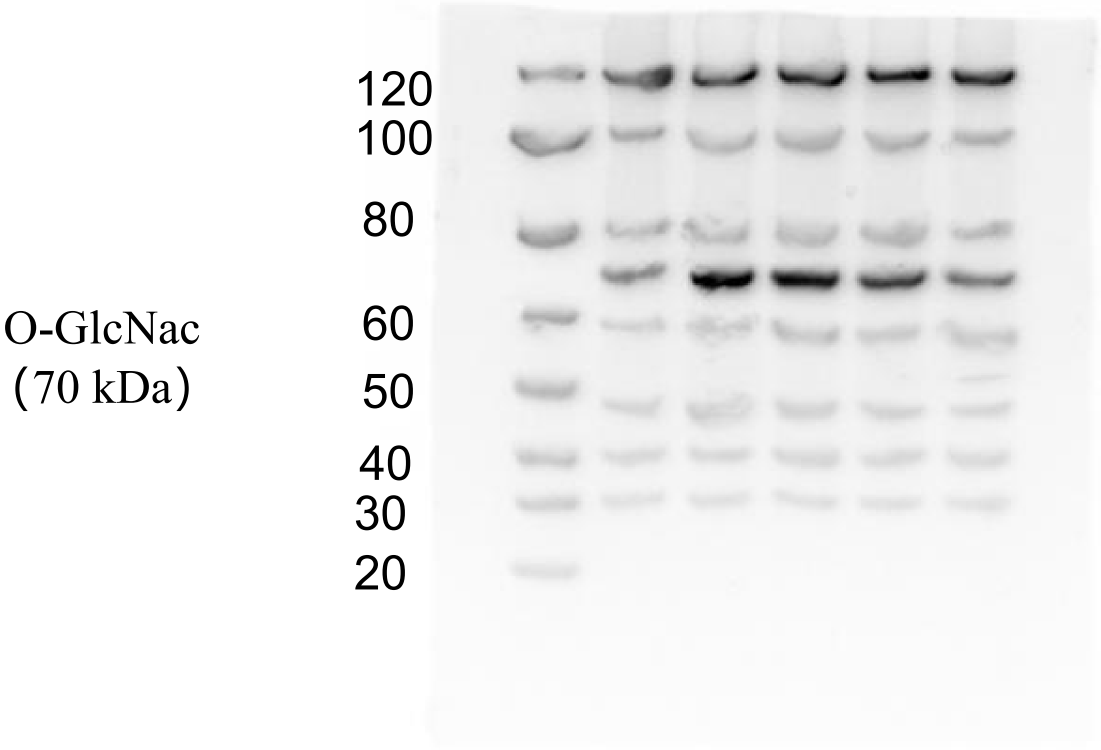
kDa
120
100
80
60
50
40
30
20
O-GlcNac
(70 kDa)

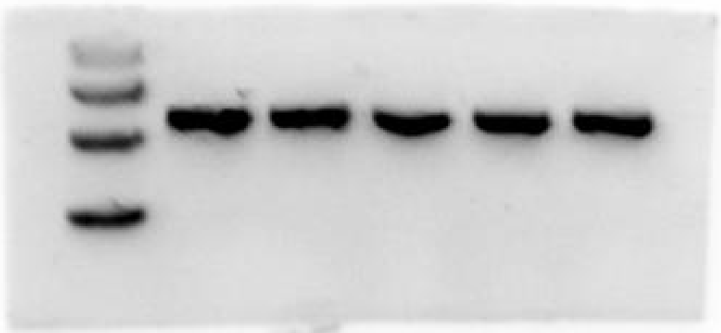
kDa
60
50
40
30
β-actin
(42 kDa)

Fig S1D

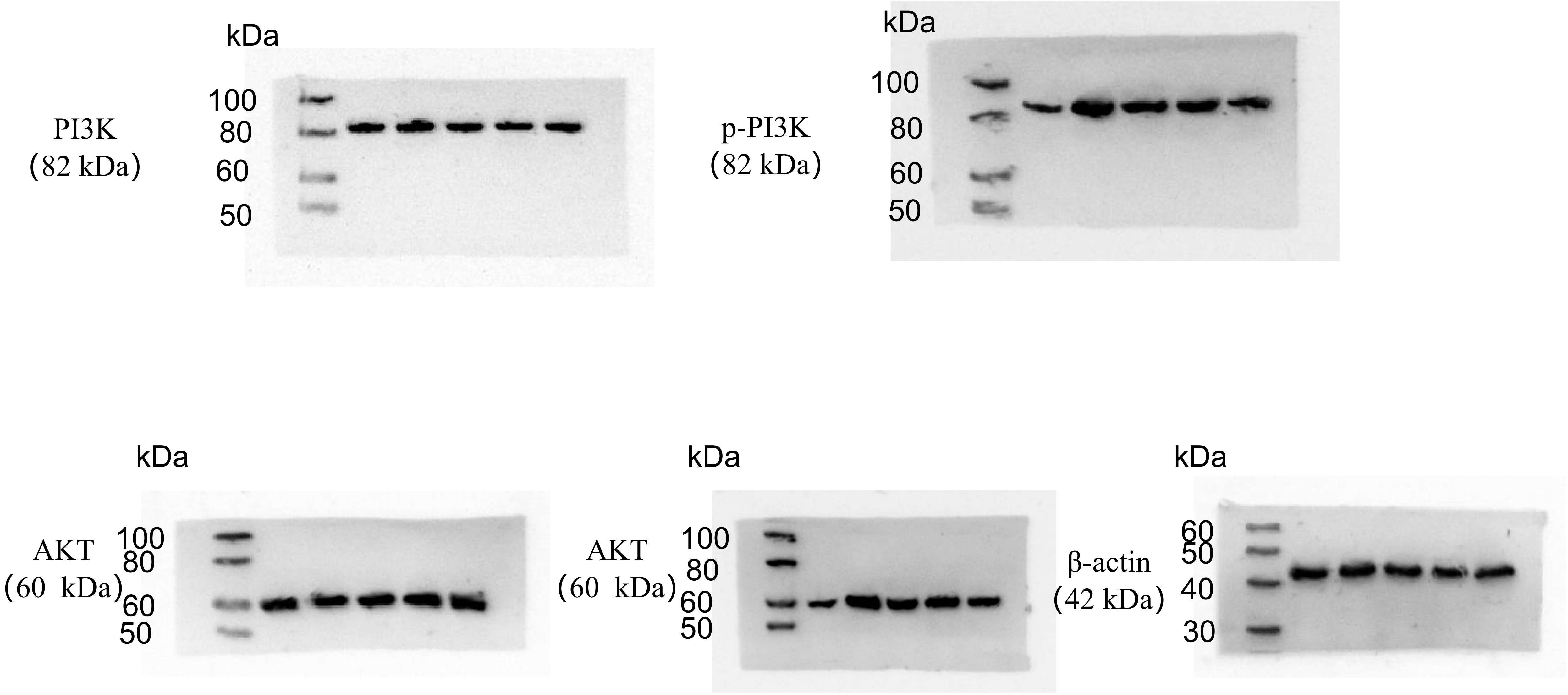
PI3K
(82 kDa)
kDa
100
80
60
50
p-PI3K
(82 kDa)
kDa
100
80
60
50
AKT
(60 kDa)
kDa
100
80
60
50
AKT
(60 kDa)
kDa
100
80
60
50
β-actin
(42 kDa)
kDa
60
50
40
30

Fig S2A

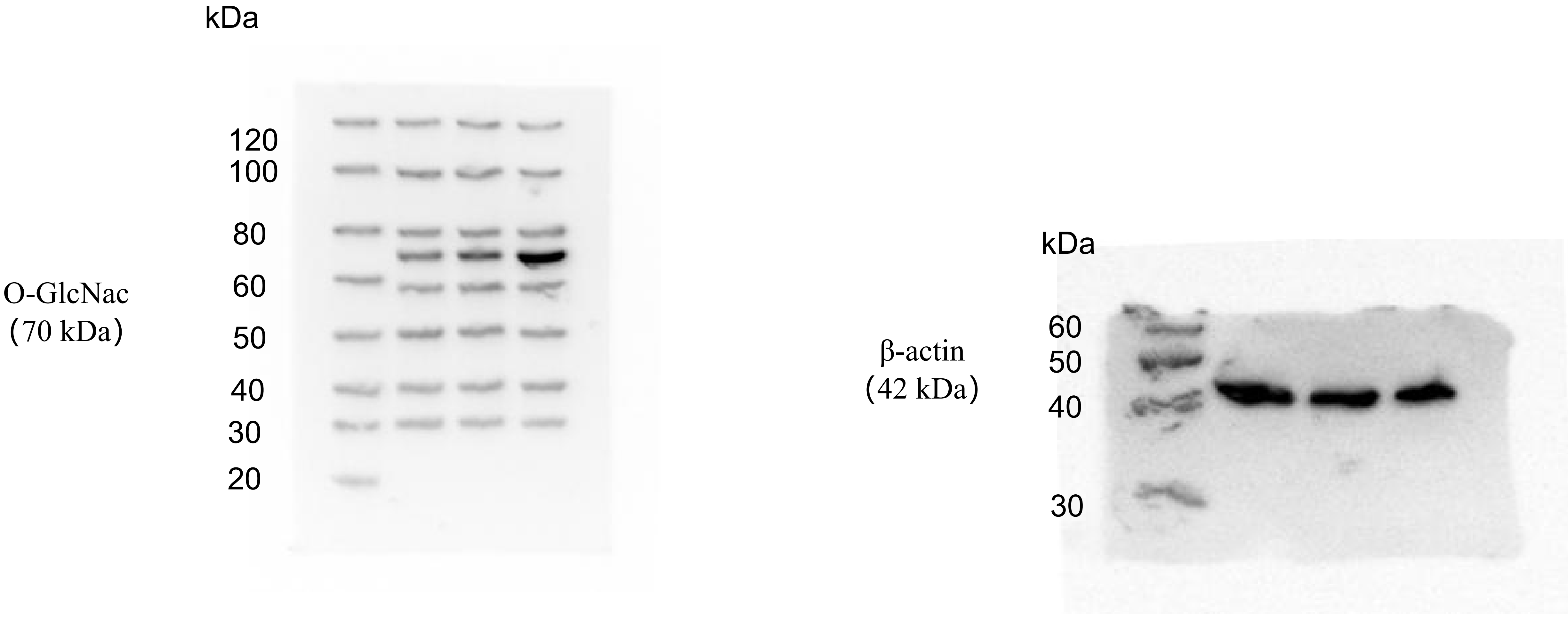
kDa
120
100
80
60
50
40
30
20
O-GlcNac
(70 kDa)
kDa
60
50
40
30
β-actin
(42 kDa)

Fig S2B

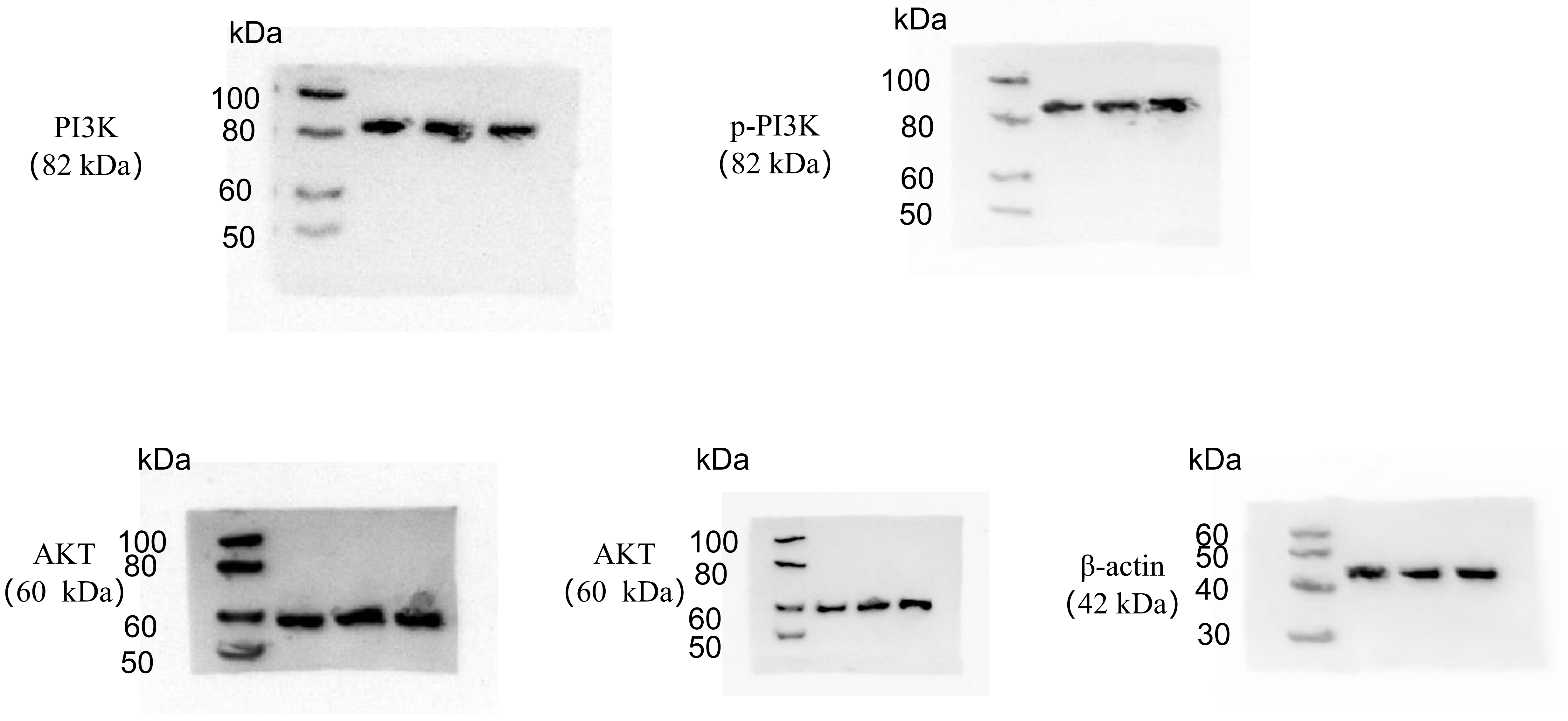
PI3K
(82 kDa)
kDa
100
80
60
50
p-PI3K
(82 kDa)
kDa
100
80
60
50
AKT
(60 kDa)
kDa
100
80
60
50
AKT
(60 kDa)
kDa
100
80
60
50
β-actin
(42 kDa)
kDa
60
50
40
30

FigS3A

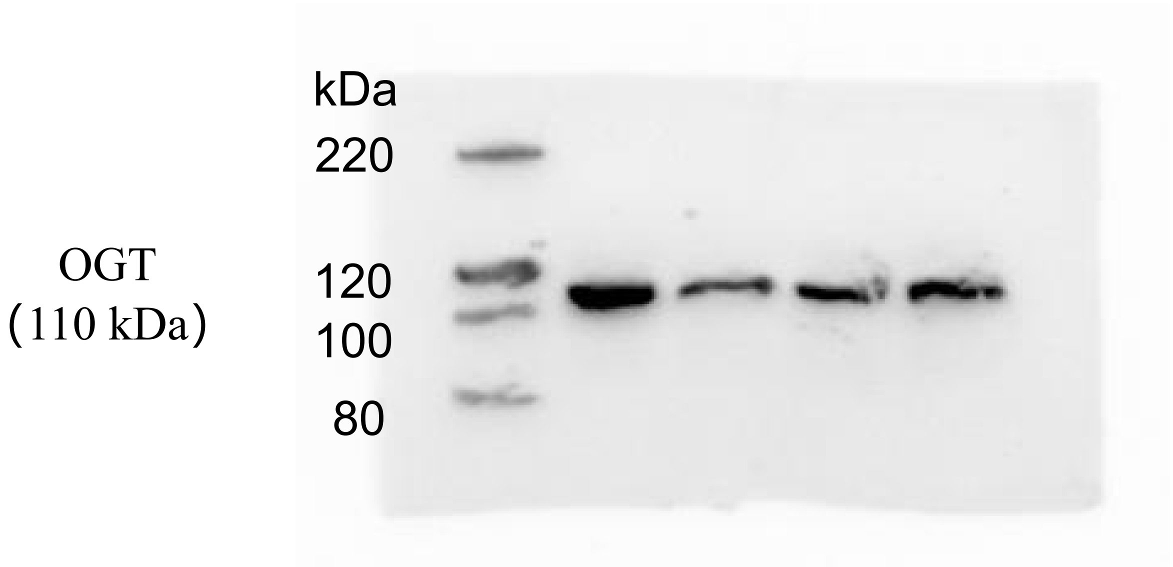

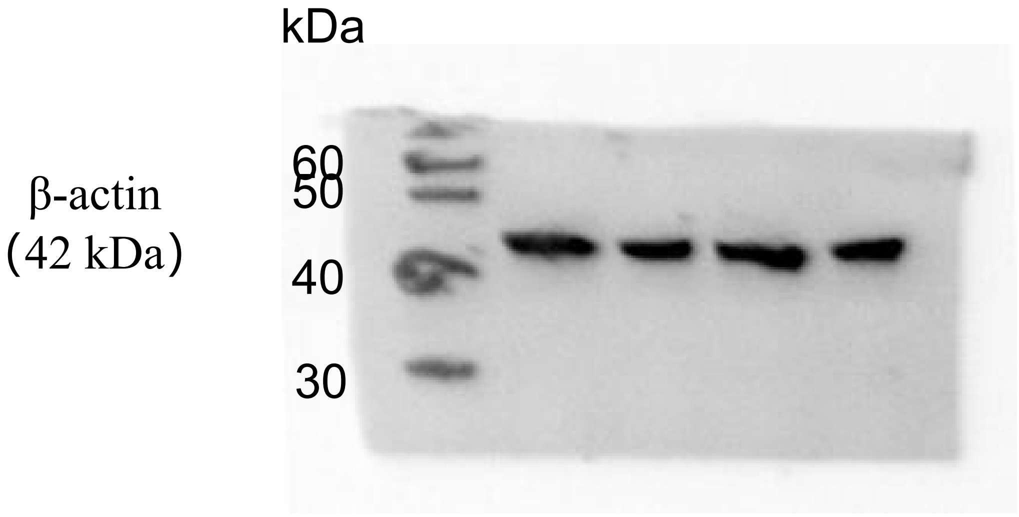

Fig S3B

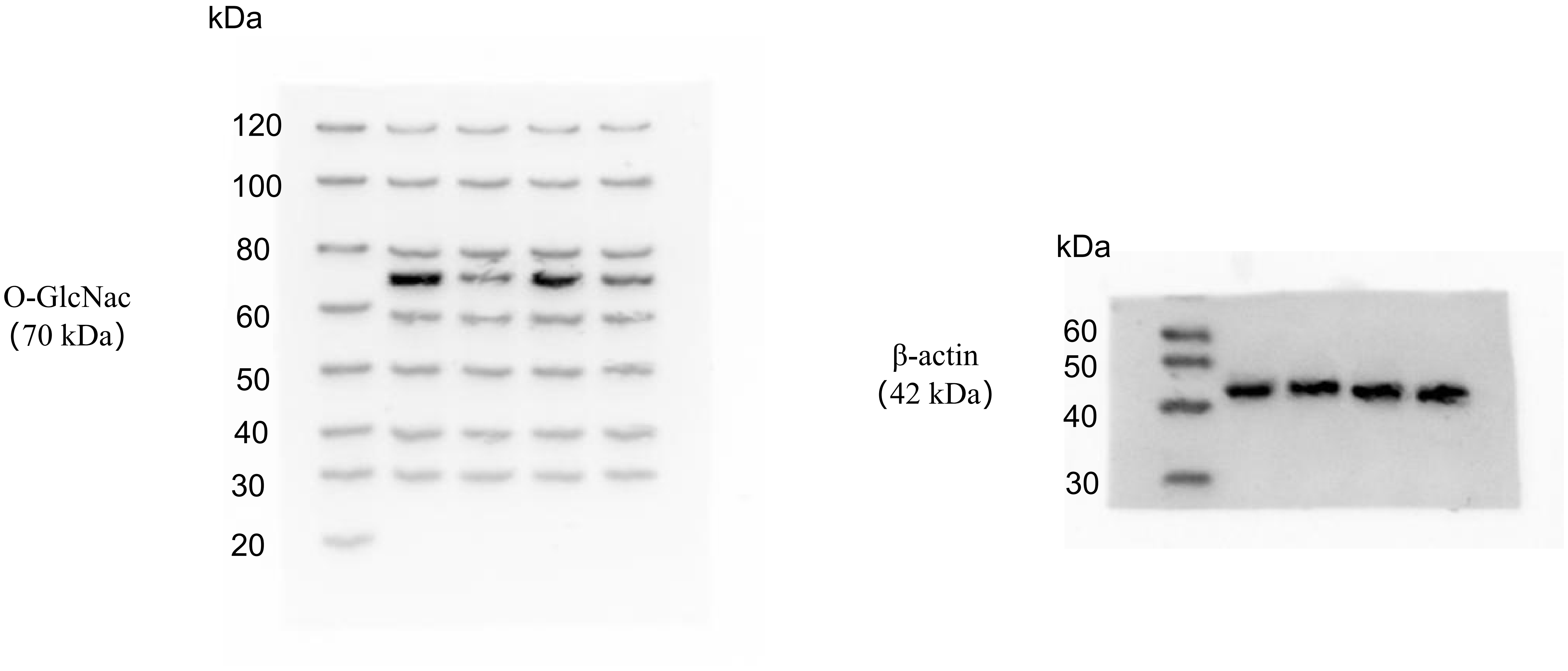
kDa
120
100
80
60
50
40
30
20
O-GlcNac
(70 kDa)
kDa
60
50
40
30
β-actin
(42 kDa)
